# Current limitations of global conservation to protect higher vulnerability and lower resilience fish species

**DOI:** 10.1038/s41598-017-06633-x

**Published:** 2017-08-09

**Authors:** Rita P. Vasconcelos, Marisa I. Batista, Sofia Henriques

**Affiliations:** MARE - Marine and Environmental Sciences Centre & FCUL - Faculdade de Ciências da Universidade de Lisboa, Campo Grande, 1749-016 Lisboa Portugal

## Abstract

Estuaries are threatened by intense and continuously increasing human activities. Here we estimated the sensitivity of fish assemblages in a set of estuaries distributed worldwide (based on species vulnerability and resilience), and the exposure to cumulative stressors and coverage by protected areas in and around those estuaries (from marine, estuarine and freshwater ecosystems, due to their connectivity). Vulnerability and resilience of estuarine fish assemblages were not evenly distributed globally and were driven by environmental features. Exposure to pressures and extent of protection were also not evenly distributed worldwide. Assemblages with more vulnerable and less resilient species were associated with estuaries in higher latitudes (in particular Europe), and with higher connectivity with the marine ecosystem, moreover such estuaries were generally under high intensity of pressures but with no concomitant increase in protection. Current conservation schemes pay little attention to species traits, despite their role in maintaining ecosystem functioning and stability. Results emphasize that conservation is weakly related with the global distribution of sensitive fish species in sampled estuaries, and this shortcoming is aggravated by their association with highly pressured locations, which appeals for changes in the global conservation strategy (namely towards estuaries in temperate regions and highly connected with marine ecosystems).

## Introduction

Estuaries are highly productive and valuable ecosystems^1^, albeit not especially diverse. But their functioning and services are threatened by continuously increasing human activities^2^ while the coherence between estuarine biota sensitivity, threats and conservation is poorly known.

Anthropogenic activities have caused loss of estuaries’ areas and connectivity with adjacent ecosystems, habitat loss (e.g. wetlands) and degradation (e.g. water quality), depletion of important species and accelerated species invasions^3^. Global fish catches in estuaries (and sea) have been increasingly dominated by less vulnerable species while more vulnerable fish became over-exploited or depleted due to “fishing down food webs” processes^4^. But disentangling human- from naturally-induced changes in estuarine biodiversity is complex since estuaries are naturally dynamic and stressed (i.e. Estuarine Quality Paradox)^5^. Estuaries are intrinsically linked with marine and freshwater ecosystems, and their fish assemblages include resident species, frequent migrants or occasional stragglers from adjacent ecosystems, as well as migratory diadromous species^6, 7^. Thus estuarine communities are potentially impacted by human activities in and around estuaries which affect species that colonize estuaries and their environmental conditions^8, 9^.

The susceptibility of a system or community to perturbation increases with an increase in its exposure and intrinsic vulnerability and with a decrease in its protection or adaptive capacity^10, 11^. Over a third of the world’s oceans show medium-high to very-high level of human activities and pressures, with coastal ecosystems (0–200 m) showing high levels of both land- and ocean-based anthropogenic activities and pressures^12^. Human-driven impacts on marine fishes include for instance lower biodiversity (i.e. taxonomic, functional and phylogenetic)^13^ and biomass^14, 15^, as well as changes in composition like less large-body and low resilience marine fishes^16, 17^. Similarly worrisome are freshwater ecosystems, with 65% of global river discharge and habitats under moderate-to-high levels of activities and pressures, explaining the global freshwater biodiversity crisis^18^. For instance, freshwater ecoregions with lower percentages of free-flowing distances show lower percentages of endemic freshwater and diadromous fishes^19^.

Despite the commitment of nations worldwide to protect ecosystems (Convention on Biological Diversity and Aichi Biodiversity Targets), and an increase in protected land and sea in the last half-century (doubling each decade)^20^, there are serious aquatic conservation shortfalls. Many protected areas exist in name only (“paper parks”) and are not (or are insufficiently) managed. Although estuaries represent most of coastal ecosystems, only a low number of estuaries and percentage of their areas is protected, and the coverage of sensitive assemblages is mostly unknown and insufficient, at least in some regions^21, 22^. Meanwhile, conservation of marine fishes (which are dominant in estuaries) is deficient, for instance marine protected areas currently provide low coverage for most species and their evolutionary history, including those with high taxonomic and functional sensitivity^10, 23, 24^.

Worldwide, estuarine fish assemblages show strong spatial patterns and environment relationships. Biogeographical region and environmental features of estuaries (e.g. temperature, connectivity and area) regulate patterns of species richness^25, 26^, composition and functional traits^27, 28^. Functional traits determine the way species use resources and their tolerance to environmental conditions. For instance, body size is a key trait as it directly relates with other traits such as mobility, trophic interactions, age at first reproduction and rate of population growth^29^. Moreover body size in aquatic ecosystems is globally unevenly distributed^27, 28, 30^. Therefore, we may also expect global patterns in such covarying traits and in the resulting species sensitivity and response to changes, which can be measured for instance with: ‘species vulnerability’ (species intrinsic extinction vulnerability to fishing)^31^, and ‘species resilience’ (species productivity or resilience to fishing)^32^ - both based on ecology and life history traits (Table 1).

**Table 1:** Description and relevance of fish traits.

Trait	Category	Description	Relevance
**‘Vulnerability’**: Intrinsic extinction vulnerability to fishing pressure	Low	0–30%	Indirect measure of species sensitivity to change, from Cheung and colleagues^31^. Based on life history and ecological features: maximum length, age at first maturity, parameter k, natural mortality rate, maximum age, geographical range, fecundity and spatial behaviour strength.
	Low to Moderate	30–40%	
	Moderate to High	40–60%	
	High to Very High	60–70%	
	Very High	70–100%	
‘**Resilience’**: Productivity or resilience to fishing pressure	High	<1.4 yr.	Indirect measure of species capacity to recover from changes in the environment from Musick and colleagues^32^, i.e. minimum population doubling time. Based on intrinsic rate of increase, von Bertalanffy growth coefficient, fecundity, age at maturity and maximum age.
	Medium	1.4–4.4 yr.	
	Low	4.5–14 yr.	
	Very Low	>14 yr.	

**Figure 1:**
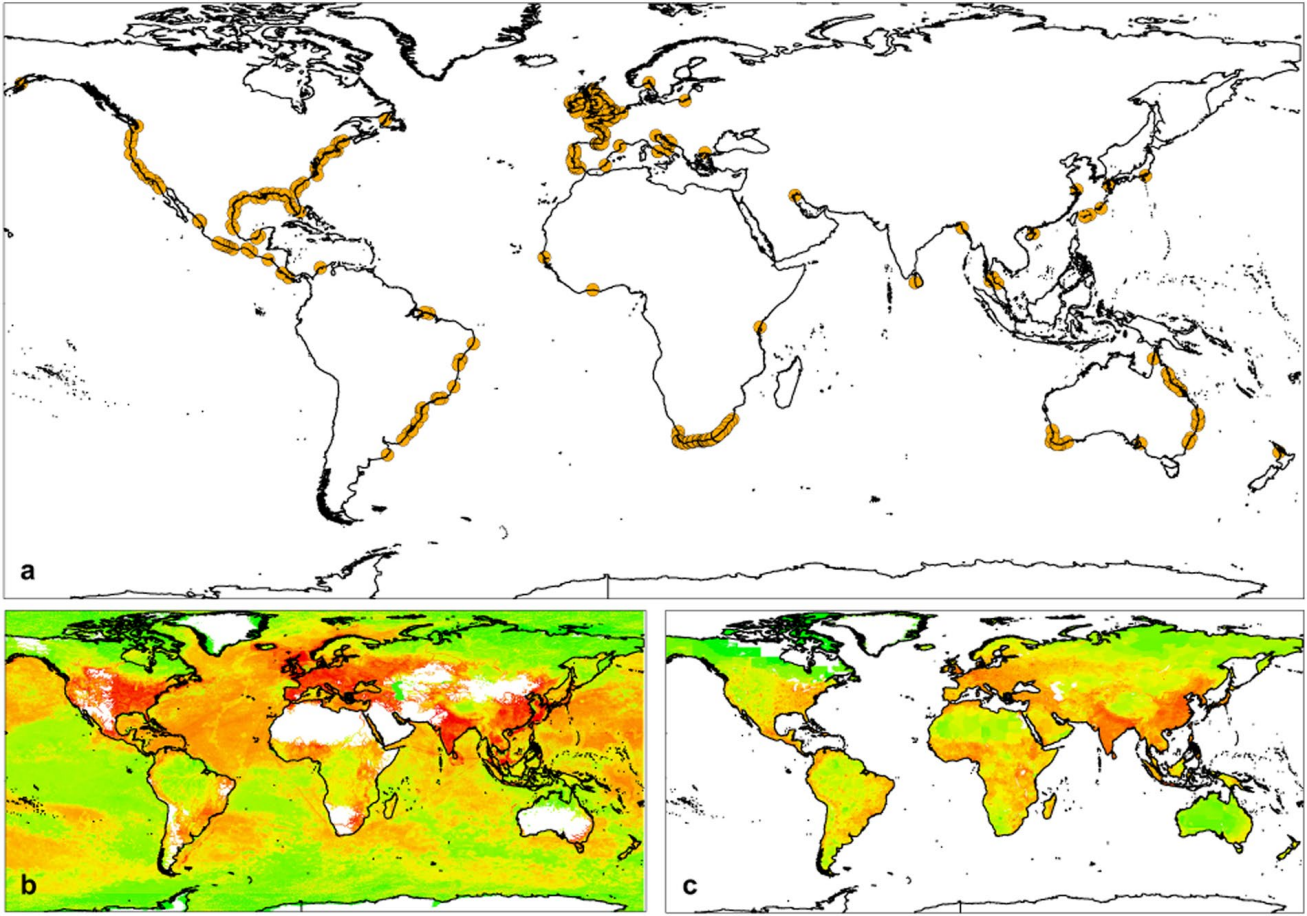
Location of estuaries included in the present study. The map was built in ArcGIS for desktop version 10.4 (http://desktop.arcgis.com). (**a**) Each estuary is represented with a circle (n = 530 samples and for 378 estuaries worldwide). (**b**) Intensity of human pressures in marine ecosystems [  between low (green) and high (red); data from Halpern and colleagues^12^ are freely available at https://www.nceas.ucsb.edu/globalmarine/data] and in freshwater ecosystems [  between low (green) and high (red); data from Vörösmarty and colleagues^18^ are freely available at http://www.riverthreat.net/]. (**c**) Human population density, which was used as intensity of human pressures in each estuary [  between low (green) and high (red); data are freely available at http://sedac.ciesin.columbia.edu/data/set/gpw-v3-population-density ^63^]. See further details in the methods section.

Here, we analyse for the first time the susceptibility of biodiversity in estuaries to stressors from multiple ecosystems, at a worldwide extent. Our aim is to identify global conservation pitfalls in estuaries by assessing whether more vulnerable and less resilient fish assemblages are associated with particular environmental features, high levels of human pressures and/or low levels of protection, potentially making them more susceptible to disturbances. To this end, using publicly available data and spatial analysis, for a set of estuaries distributed worldwide (Fig. 1) we characterized the fish assemblages (Supplementary methods and Table S1), the vulnerability and resilience traits of their species (Table 1) as well as ecosystem features (Supplementary methods and Table S2). We also estimated intensity of human activities and pressures, as well as protection in and around each estuary (i.e. for marine, estuarine and freshwater ecosystems due to their inherent connectivity; but separately per ecosystem). Finally, we used correlations, linear- and linear mixed models to: identify links between environmental conditions, human pressures and protection worldwide; and to assess if more vulnerable and less resilient assemblages occur in estuaries with particular conditions and if they are exposed to low levels of protection and high levels of exposure to human pressure worldwide.

## Results and Discussion

**Figure 2:**
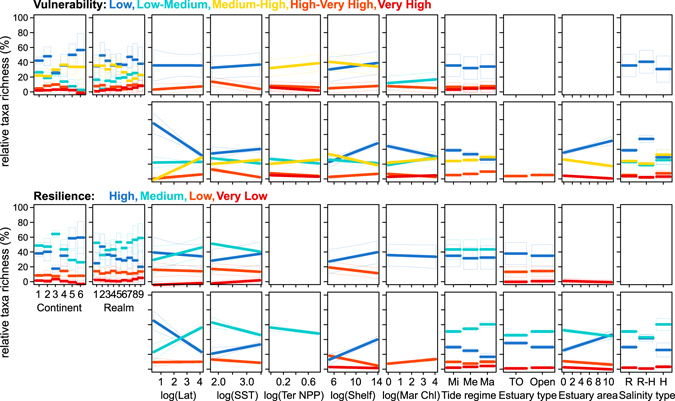
Relationships between fish traits (i.e. relative taxa richness of trait categories) of fish assemblages in sampled estuaries distributed worldwide and ecosystem features, according to fitted linear models. Traits considered are vulnerability, i.e. species intrinsic extinction vulnerability to fishing      Low (dark blue), Low-Medium (light blue), Medium-High (yellow), High-Very High (orange), Very High (red)﻿ and resilience, i.e. species productivity or resilience to fishing     High (dark blue), Medium (light blue), Low (orange), Very Low (red). Ecosystem features represented are: continent (1- North America, 2- South America, 3- Europe, 4- Africa, 5- Asia, 6- Oceania), marine biogeographical realm (1- Temperate Northern Pacific, 2- Tropical Eastern Pacific, 3- Temperate South America, 4- Temperate Northern Atlantic, 5- Tropical Atlantic, 6- Temperate Southern Africa, 7- Western Indo-Pacific, 8- Central Indo-Pacific, 9- Temperate Australasia), latitude (for representation purposes only), sea surface temperature (SST), terrestrial net primary productivity (Ter NPP), marine chlorophyll a (Mar Chl), continental shelf width (Shelf), tidal regime (Mi-microtidal, Me-mesotidal, Ma-macrotidal), estuary type (TO-temporarily open, O-open) and salinity type (R-regular, R-H-regular to hyperhaline, H-hyperhaline). Only predictors with relative importance above 0.5 in linear models are represented.

**Figure 3:**
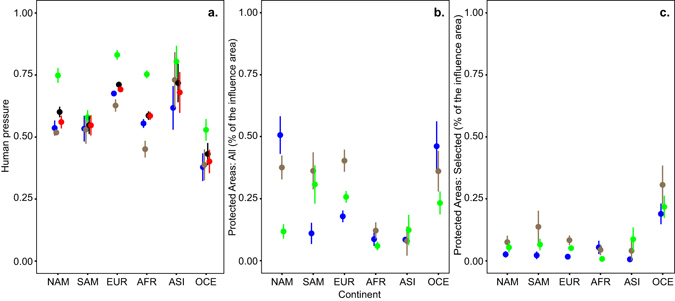
Mean (±confidence interval of 95%) per continent (NAM- North America, SAM- South America, EUR- Europe, AFR- Africa, ASI- Asia, OCE- Oceania) of: exposure of sampled estuaries to human activities and pressures (**a**), as well as percentage of coverage of those estuaries by protected areas (**b**) and by selected protected areas with IUCN I-IV categories (**c**). These three aspects were measured directly for:    the estuary (brown), the adjacent coastal marine ecosystem (blue) and the adjacent freshwater ecosystem (green). In addition, human activity and pressure is also represented as:   the mean of the three ecosystems (black), and the weighted mean of the three ecosystems (red; where, for each estuary, the weight of each ecosystem is given by the percentage of taxa from that ecosystem in the estuarine assemblage. Globally, higher intensity of human activities and pressures are found in sampled estuaries of Asia and Europe and lower in Oceania, regardless of the ecosystem of influence considered (marine, estuary, freshwater). Percentage of protected area (by selected PA with IUCN I-IV categories) is higher in sampled estuaries of Oceania.

Briefly, this study shows that the vulnerability and resilience traits of fish from the sampled estuarine assemblages worldwide are particularly associated with geography and certain environmental gradients (Fig. 2; Table 2). Additionally, in short, exposure to human driven pressures and extent of protection in sampled estuaries are inversely related and are also heterogeneously distributed across the globe (Figs 1 and 3; Table 3), with many estuaries with intense human pressure supporting more vulnerable and less resilient fish species, despite no strong relation with extent of protection (Fig. 2; Table 4). In all, results provide insight into global conservation needs of estuaries based on the vulnerability and resilience of their fish assemblages.

**Table 2:**
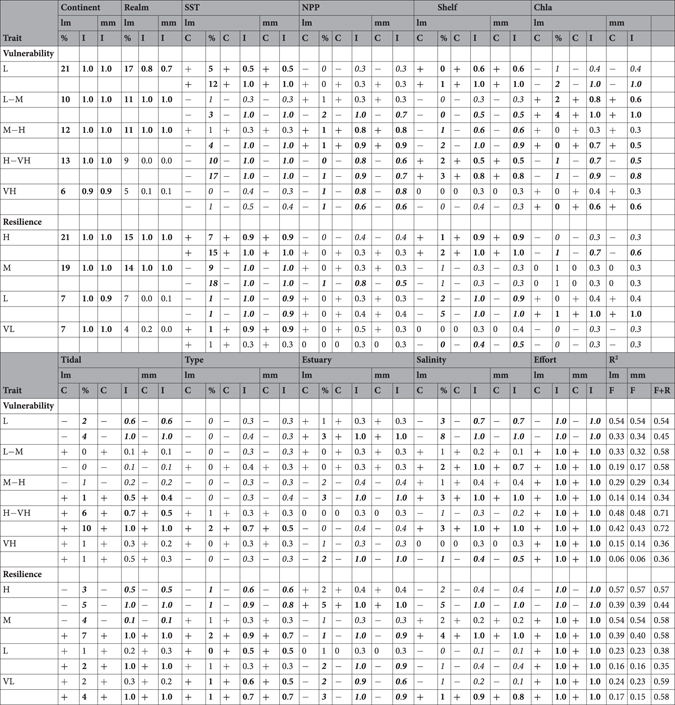
Effect of ecosystem features (in columns) on “relative taxa richness” of fish vulnerability and resilience (in rows) among estuaries distributed worldwide, according to the fitted linear models (lm) and linear mixed models (mm).

Fish vulnerability categories are: low (L), low to moderate (L-M), moderate to high (M-H), high to very high (H-VH) and very high (VH). Fish resilience categories are: high (H), medium (M), low (L) and very low (VL). For each trait category, we built two alternative models (in rows): with and without biogeographic variables (respectively, upper and lower row). To explore lm and mm, we used a multimodel procedure: the table shows the predictor coefficient in lm (represented in the table as C, shown only as “+” if positive or “−” if negative; R package relaimpo), the importance of each predictor to deviance in lm (represented in the table as %, between 0–100%; R package relaimpo), and the relative importance of each predictor to trait variation in lm and mm (represented in the table as I, between 0–1; package MuMIn). The table also shows: for lm, the pseudo R^2^ of the fitted lm; for mm, the conditional pseudo R^2^ (fixed effects) and the marginal R^2^ (fixed and random effects). Ecosystem features are: continent and marine biogeographic realm, sea surface temperature, terrestrial net primary productivity, continental shelf width, marine chlorophyll a, tidal regime (from microtidal, mesotidal, to macrotidal), estuary type (from temporarily open to open), estuary area, salinity type (from regular, regular-hyperhaline to hyperhaline) and sampling effort (in total sampled area). Continuous predictors were log-transformed. Fish traits are species intrinsic vulnerability and resilience (total number of samples is 530, for a total of 378 estuaries). Values in italic font are predictors that have C (coefficient) below 0, and values in bold font are predictors that have I (importance) above 0.5.

**Table 3:** Pairwise Pearson correlation between intensity of human pressure (H), percentage of coverage by protected areas (PA), percentage of coverage by selected areas of IUCN management categories I-IV (PAS), area used for estimation of pressure and protection (A, in km^2^), and environmental variables in and around estuaries distributed worldwide.

	Human pressure					Protection - All			Protection - Selected			Area			Environmental										
	H_mar_	H_est_	H_fre_	H_mean_	H_wmean_	PA_mar_	PA_est_	PA_fre_	PAS_mar_	PAS_est_	PAS_fre_	A_mar_	A_est_	A_fre_	Lat	SST	NPP	She	Chla	Tid	Typ	Mou	Est	Bas	Sal
H_mar_																									
H_est_	**0**.**5**																								
H_fre_	0.4	0.4																							
H_mean_	**0**.**8**	**0**.**8**	**0**.**7**																						
H_wmean_	**0**.**9**	**0**.**7**	**0**.**5**	**0**.**9**																					
PA_mar_	−*0*.*3*	−*0*.*1*	ns	−*0*.*2*	−*0*.*3*																				
PA_est_	ns	−*0*.*1*	ns	−*0*.*1*	−*0*.*1*	**0**.**6**																			
PA_fre_	ns	ns	−*0*.*3*	−*0*.*1*	ns	0.1	**0**.**5**																		
PAS_mar_	−*0*.*2*	−*0*.*2*	−*0*.*3*	−*0*.*3*	−*0*.*3*	0.4	0.2	0.1																	
PAS_est_	−*0*.*3*	−*0*.*3*	−*0*.*3*	−*0*.*4*	−*0*.*3*	0.3	**0**.**6**	0.3	**0**.**6**																
PAS_fre_	−*0*.*2*	−*0*.*2*	−***0***.***5***	−*0*.*4*	−*0*.*3*	0.2	0.3	**0**.**6**	0.3	**0**.**5**															
A_mar_	−*0*.*2*	ns	ns	ns	−*0*.*1*	0.2	0.4	0.4	−*0*.*1*	0.2	0.2														
A_est_	ns	0.1	ns	ns	ns	0.4	0.2	0.1	ns	−*0*.*1*	ns	0.3													
A_fre_	ns	0.1	0.1	0.1	ns	0.2	0.1	ns	ns	ns	ns	0.3	**0**.**6**												
Lat	0.4	ns	0.4	0.3	0.4	ns	0.1	ns	−*0*.*1*	ns	ns	0.2	−*0*.*1*	ns											
SST	−***0***.***5***	−*0*.*1*	−*0*.*2*	−*0*.*3*	−***0***.***5***	ns	−*0*.*3*	−*0*.*2*	0.2	ns	ns	−*0*.*3*	ns	−*0*.*1*	−***0***.***7***										
NPP	ns	ns	ns	ns	ns	−*0*.*1*	ns	ns	ns	ns	ns	ns	ns	ns	ns	0.1									
She	−*0*.*2*	ns	0.1	ns	−*0*.*1*	0.2	0.4	0.3	ns	0.2	0.2	**1**.**0**	0.2	0.2	0.2	−*0*.*3*	ns								
Chla	ns	ns	0.3	0.1	ns	0.2	0.2	ns	−*0*.*2*	ns	−0.1	**0**.**5**	0.3	0.3	0.2	−*0*.*3*	−*0*.*2*	**0**.**5**							
Tid	0.2	0.2	0.1	0.2	0.2	0.2	0.4	0.3	ns	0.2	0.1	**0**.**5**	ns	ns	0.2	−***0***.***5***	−*0*.*1*	**0**.**5**	0.2						
Typ	0.1	0.3	ns	0.2	ns	0.3	0.3	0.3	ns	ns	0.2	0.4	0.4	0.4	ns	−*0*.*3*	−*0*.*1*	0.3	0.2	0.4					
Mou	ns	0.1	ns	0.1	ns	0.4	0.4	0.2	ns	ns	0.1	**0**.**5**	**0**.**7**	**0**.**5**	ns	−*0*.*3*	ns	0.4	0.4	0.4	**0**.**5**				
Est	ns	0.2	ns	0.1	ns	0.4	0.3	0.2	ns	ns	0.1	0.4	**0**.**9**	**0**.**7**	−*0*.*1*	−*0*.*1*	ns	0.3	0.3	0.1	**0**.**5**	**0**.**8**			
Bas	ns	0.1	0.1	0.1	ns	0.2	0.2	0.1	ns	ns	ns	0.3	**0**.**6**	**0**.**9**	ns	−*0*.*1*	ns	0.2	0.3	ns	**0**.**5**	**0**.**5**	**0**.**7**		
Sal	−*0*.*3*	ns	−*0*.*1*	−*0*.*2*	−*0*.*3*	ns	ns	ns	0.1	0.2	0.1	ns	ns	ns	−*0*.*1*	0.2	−*0*.*2*	ns	ns	−*0*.*1*	ns	ns	ns	ns	

Each variable about human pressure and protection was estimated for marine (mar), estuarine (est) and freshwater ecosystems (fre); and mean and weighted mean (wmean) of human pressure are also included. Continuous environmental variables are: Lat - latitude, SST - sea surface temperature, Ter NPP - terrestrial net primary productivity, Mar Chl - marine chlorophyll a, She - continental shelf width, Tid - tidal regime, Typ - estuary type, Mou - estuary mouth width, Est - estuary area, Bas - drainage basin area, Sal - salinity type. All variables were log transformed (except tidal regime, estuary type and salinity type) and in addition pressure variables were normalized (scaled to vary between 0 and 1). ns - not significant at p < 0.05. (Total number of samples is 530, for a total of 378 estuaries). Correlations below 0 are in italic, and correlations above 0.5 or below −0.5 are in bold font.

**Table 4:** Pairwise Pearson correlation between traits of the fish assemblages (relative taxa richness of trait categories) in a set of estuaries distributed worldwide with: intensity of human pressure (H), percentage of coverage by protected areas (PA) and percentage of coverage by selected areas of IUCN management categories I-IV (PAS).

Trait	Category	Human pressure					Protection - All			Protection - Selected		
		H_mar_	H_est_	H_fre_	H_mean_	H_wmean_	PA_mar_	PA_est_	PA_fre_	PAS_mar_	PAS_est_	PAS_fre_
Vulnerability	Low (L)	−***0***.***5***	−*0*.*3*	−*0*.*4*	−***0***.***5***	−***0***.***5***	ns	−*0*.*1*	ns	0.2	0.2	0.3
	Low−Moderate (L-M)	0.2	0.2	0.2	0.2	0.2	0.1	0.2	0.1	−*0*.*1*	ns	−*0*.*1*
	Moderate-High (M-H)	0.3	0.1	0.3	0.3	0.3	−*0*.*2*	ns	ns	−*0*.*1*	−*0*.*1*	−*0*.*2*
	High-Very High (H-VH)	0.4	0.2	0.2	0.3	0.4	ns	0.2	0.2	−*0*.*2*	ns	ns
	Very High (VH)	ns	ns	0.2	0.1	0.1	−*0*.*1*	ns	ns	−*0*.*1*	−*0*.*1*	−*0*.*2*
Resilience	High (H)	−***0***.***5***	−*0*.*3*	−*0*.*4*	−***0***.***5***	−***0***.***5***	0.1	ns	−*0*.*1*	0.1	0.2	0.2
	Medium (M)	0.4	0.3	0.3	0.4	0.4	ns	0.2	0.2	−*0*.*1*	ns	ns
	Low (L)	0.1	ns	0.1	0.1	0.2	ns	ns	ns	−*0*.*1*	−*0*.*2*	−*0*.*2*
	Very Low (VL)	0.2	0.2	0.2	0.3	0.2	−*0*.*1*	ns	ns	−*0*.*1*	−*0*.*1*	−*0*.*1*

Each variable for human pressure and protection was estimated for marine (mar), estuarine (est) and freshwater ecosystems (fre); and mean and weighted mean (wmean) of human pressure are also included. All variables (except fish traits) were log transformed and in addition pressure variables were normalized (scaled to vary between 0 and 1). ns - not significant at p < 0.05. (Total number of samples is 530, for a total of 378 estuaries). Correlations below 0 are in italic, and correlations above 0.5 or below −0.5 are in bold font.

Results show that human pressure directly in sampled estuaries (H_estuary_) and in the adjacent marine and freshwater ecosystems (H_marine_, H_freshwater_) are moderately correlated (pairwise Pearson correlations 0.38–0.52), and strongly correlated with mean intensity from these three ecosystems (pairwise Pearson correlations 0.73–0.84 with H_mean_, and 0.54–0.95 with H_weighted-mean_) (Fig. 1; Table 3). It is acknowledged that human pressures are intense in coastal zones, especially near heavily populated zones^33–36^, and estuaries are directly exposed due to location and with their surrounding ecosystems impacted by cumulative aggregated activities^12, 37^. The data used represent a set of human activities and human-induced pressures, therefore they only indirectly inform on human-induced impacts, due to possibly different mitigation measures and local context. The current analysis could benefit from a higher spatial resolution of data on human pressures^38^, but such data are not available for all regions across this global extent. Still, our results indicate that the fish assemblage in a given estuary tends to receive a similar degree of exposure to human pressures from these three ecosystems. This represents an added challenge to the already complex conservation and spatial planning of estuaries^9^, namely in scenarios with high pressures in estuary and surrounding ecosystems.

Furthermore, our results also indicate that existing conservation efforts in sampled estuaries are partially related with those in the surrounding ecosystems: i.e. the extent of coverage by protected areas (PA) from the three ecosystems is moderately correlated (pairwise Pearson correlations 0.11–0.60 for PA_all_ and 0.32–0.57 for selected PA_I–IV_, lower between marine and freshwater ecosystems) (Table 3). The observed relation could be generated by a consistency of conservation policies: the level of conservation policies/investments in a given country/region/continent seems roughly similar across ecosystems, which can be due to socio-economic and political context. Still, potential protection should not be confounded with realized protection. Benefits from marine protection are acknowledged to depend on protection level and effectiveness which is influenced by factors including enforcement, stakeholders engagement, presence of no-take zones, surrounding human pressure, size, isolation and age^39, 40^. Furthermore, connectivity and interactions across the marine-estuarine-freshwater gradient should be central in conservation planning, since estuaries are known to assemble fish species from these ecosystems (marine species often dominant^6, 7^) with connectivity essential for maintaining species life cycles and ecosystem functioning^9, 41, 42^. But despite increasing management plans that include continuous protected areas across more than one ecosystem, conservation across land-river-marine realms and across freshwater-estuarine-marine realms are insufficiently applied^36, 42^.

In the set of sampled estuaries, there is a decoupling of the coverage by protected areas and intensity of human pressure: within each ecosystem, there is −0.14 to −0.49 pairwise Pearson correlation between protection and human pressure (Table 3). But this global negative relationship between protection and human pressure is not present in all continents, it arises amid considerable variability (Supplementary Fig. S1) and may be due to the current set of analysed estuaries - since different continents show disparate intensity of human pressures and extent of coverage by protected areas (Fig. 3), distribution and number of samples (due to uneven distribution of adequate fish assemblage data for this analysis; Supplementary Table S2). This advises caution in interpretation of the obtained global patterns. Still, the sampled estuaries covered all continents and the full spectrum of pressure intensity (from very low to very high as described in the work by Halpern and colleagues for marine ecosystems^12^) reinforcing the present results. Indeed, it is recognized that many protected areas are placed in zones with intense human pressures^33, 35, 36^, but large marine and land reserves are also often strategically placed in zones where conflicts with multiple human activities are minimized in advance (such as marine offshore zones, or higher and unproductive lands), which might decrease effectiveness of protected areas^43, 44^. Nevertheless, it has been previously shown that coastal marine reserves (usually more exposed to human threats) are as effective in protecting biodiversity as those placed offshore or in less-developed locations^43^, therefore protecting locations highly exposed to human pressures should be pursued in a global conservation strategy.

Intensity of human pressures in and around sampled estuaries shows a clear geographical pattern (higher in Europe and Asia, intermediate in Africa, North America and South America, and lower in Oceania), regardless of the variable considered (H_marine_, H_estuary_, H_freshwater_, H_mean_ or H_weighted-mean_) (Fig. 3). This pattern reflects the mean human population density of continents (http://data.worldbank.org). Meanwhile, percentage of coverage by protected areas is higher in sampled estuaries of Oceania and lower in Africa and Asia. The difference between estuaries in different continents observed here reflects the known vastly divergent protection regimes for marine ecosystems (0–200 nautical miles) implemented in those continents (with Oceania notably standing out), and are less akin to terrestrial protection (which is broader in Central and South America)^20^. Here, the difference in protection of sampled estuaries between continents is especially evident for protected areas of I–IV IUCN management categories (selected PA_I–IV_), which are known to represent approximately 25–55% of protected areas in all continents, but dominate in Oceania (around 85%) and are scarce in Africa (around 10%)^20^. It is widely acknowledged that despite efforts to reach conservation targets (10% of sea and 17% of land by 2020 - Convention on Biological Diversity), efforts are geographically imbalanced globally, and coverage by marine no-take areas is reduced (0.08%) and many or even most protected areas are inadequately managed, lacking an integrative network design^45^.

Overall, there are contrasting scenarios of protection and human pressures in sampled estuaries across the globe, notably (Fig. 3): a) high human pressure and medium protection of estuarine fish assemblages in Europe (high coverage by PA_all_, but low coverage by selected PA_I–IV_); b) high pressure and low protection of estuarine fish assemblages in Asia; c) low pressure and high protection of estuarine fish assemblages in Oceania. The low coverage of sampled estuaries by protected areas with stricter measures (PA_I–IV_) observed in most continents, especially in estuaries with intense human pressure, highlights a likely conservation shortfall regarding many estuarine fish assemblages (as seen in fish assemblages of other aquatic ecosystems^10, 24^), and argue in favour of urgently revising management and conservation plans.

In our dataset, estuaries in higher latitudes (and with lower temperature) tend to have higher intensity of human pressure, but the latitude cline is weakly and ambiguously related with the coverage by protected areas (Table 3). The observed latitudinal increase in human pressure in and around estuaries resembles the reported latitudinal increase in GDP per capita, but contrasts with the acknowledged latitudinal decrease in population density^46^. Nevertheless, this latitudinal trend should be viewed with caution - in our study, the location of sampled estuaries is imposed by fish assemblage data, and scarce data on fish assemblages in some regions results in a smaller representation of such regions (e.g. tropical and subtropical Asia where human pressure is often high, and some very high latitude regions where human pressure is low^12^); whereas human pressures in rapidly developing regions may be underestimated. Additionally, results show that sampled estuaries with higher connectivity with the marine ecosystem (tidal regime, estuary type, mouth width) and larger area (of the estuary and drainage basin) tend to have higher human pressure (especially H_estuary_), likely because they attract larger human populations, but they also have higher protection (when considering PA_all_), possibly due to higher conservation obligations.

Fish species ‘vulnerability’ and ‘resilience’ traits are inversely correlated in the surveyed estuaries (Table S3), since both are based on life history and ecological characteristics (with four shared parameters)^31, 32^. Moreover, body size is used to parameterize vulnerability, and several parameters in vulnerability and resilience are acknowledged to covary with size^29^, resulting in size, here, being positively correlated with vulnerability and negatively with resilience (Table S3). For instance, geographical range size of marine fishes has been shown to increase with adult (e.g. size, schooling behaviour) and larval traits (e.g. pelagic larval duration) that together affect dispersal and post-dispersal persistence of new populations^30, 47^. Similarly, larger species have been reported to have higher fecundity, older maximum- and first maturity-age [also lower von Bertalanffy growth coefficient (K)] and slower intrinsic population growth rate^29, 48^.

Sampled estuaries worldwide are on average dominated by species with low vulnerability, and high to medium resilience (Table 5) (and concurrently by species with small to medium maximum size^27^). Accordingly, it has been shown that abundance decreases with the increase in body mass for trophic webs generally^29^ and that opportunistic and periodic life-history strategies dominate in European estuaries, where equilibrium strategy is rarer (large generation time and age-specific survivorship, small fecundity, chiefly marine stragglers)^28^.

**Table 5:** Relative taxa richness (%; mean and standard deviation) of fish vulnerability and resilience traits among estuaries distributed worldwide.

Trait	Category	Mean	SD
Vulnerability	Low	40	19
	Low-Moderate	22	9
	Moderate-High	23	11
	High-Very High	6	7
	Very High	4	4
Resilience	High	32	19
	Medium	50	16
	Low	8	7
	Very Low	2	3

(Total number of samples is 530, for a total of 378 estuaries).

Moreover, vulnerability and resilience traits differ among sampled estuaries globally, and their distributions relate with environmental conditions (as shown with linear- and linear mixed models) and with human pressures (as shown with Pearson correlation). Explicitly, opposite relationships are evident in fishes with different degrees of vulnerability (namely low versus other higher categories); as well as in fishes with different degrees of resilience (namely high versus other lower categories) (Fig. 2; Table 2). The proportion of fishes with low vulnerability (and high resilience) decreases in estuaries in Europe, and markedly in estuaries that are from higher latitudes (lower temperatures) and that have higher connectivity with the marine ecosystem (wide tidal amplitude, and in permanently open estuaries) (Fig. 2; Table 2). Meanwhile, the inverse spatial pattern and trait-environment relationship occurs for fishes with higher vulnerability (and lower resilience) (Fig. 2; Table 2). This global pattern of vulnerability and resilience mirrors the global pattern previously observed for body size in these estuaries^27^ due to trait covariation. Several mechanisms have been proposed for the latitudinal and temperature cline in marine fishes body size^49^, including energetic and biotic advantage of smaller fish at higher temperature versus larger fishes at lower temperatures^30^. Simultaneously, it is known that estuaries with less connectivity with the marine ecosystem hinder colonization by marine fishes^50^ (which tend to be larger than freshwater fishes in this database^27^, with both marine and freshwater fishes in European estuaries previously reported as larger than residents^28^). The observed link (of fish vulnerability and resilience with environmental conditions in estuaries worldwide) is further supported by previous regional evidence that life-history strategies relate with environmental conditions in estuaries and river basins, these strategies being consistent with climate regime and historical events (chiefly stability of suitable conditions)^28, 51^. Overall, present results should be seen as a first attempt to identify the main current conservation concerns for estuaries at a global extent, although the observed trait patterns and trait-environment relationships might be influenced by some data limitations (i.e. spatial differences in availability of assemblage studies, and of survey sampling method, effort and coverage of estuarine habitats). Still, the observed patterns seem broadly supported by their compliance with above-mentioned previous studies.

The currently known global pattern of fish body size in estuaries is based on inter-species variability^27^, but analysing intra-species variability (i.e. size-frequencies per estuary) would expand knowledge of trait-environment relationships, since estuaries are typically nurseries. Additionally, vulnerability and resilience traits measured here are species-specific^30, 31^ but developing size-specific vulnerability traits would allow considering influence of life-stage and size on response to disturbances.

Our approach revealed that intense anthropogenic pressures in and around sampled estuaries overlap many estuarine fish assemblages with higher sensitivity traits, and this occurs in: estuaries in particular regions (high latitude, especially Europe) and estuaries with certain environmental features (high connectivity with the marine ecosystem - open and with wide tidal amplitude), as modelled (Fig. 2; Table 2). Sampled estuaries with greater human pressure tend to have species with higher vulnerability and lower resilience (Fig. 2; Table 4). This overlap raises some global conservation concern, especially since in and around the sampled estuaries the percentage of coverage by protected areas slightly tends to decrease with the increase of human pressure (Table 3), but is poorly related with assemblage sensitivity (Fig. 2; Table 4). Also concerning is the small percentage of coverage provided by protected areas with IUCN management categories I-IV (PA_I-IV_, that restrict human activities and more likely benefit biodiversity) in sampled estuaries of most regions - except Oceania. A mismatch between protected areas and desirable conservation, aiming at preserving global fish biodiversity, has been also reported for other aquatic ecosystems. For instance, there is poor protection of distribution range of most marine species^24^, and of impacted marine zones with high endemism^52^, biodiversity^35^ or high taxonomic and functional sensitivity (although not species rich)^10^.

Taxonomic biodiversity, especially hotspots^52, 53^ is prominent in conservation since it is acknowledged that maintaining high species richness expectedly improves community resilience to environmental stress, and conserving endemism presumably safeguards genetic variability, with both likely preventing biodiversity erosion^52, 54^. In contrast, little attention is given in conservation to traits and functional diversity, despite their role in maintaining ecosystem functioning and stability^55^. Although estuaries are not typically highly taxonomically diverse, they support high productivity and ecosystem services^1, 2, 9^, and therefore have high conservation value. Globally, estuarine fish species richness is known to increase towards the equator (which is a general ecological rule^56^) and in open systems^57^, advocating the conservation value of systems with those characteristics. Present results reinforce the value of estuaries with high connectivity with the marine ecosystem and in addition support the conservation value also of temperate estuaries, as species in those systems have higher vulnerability and lower resilience. Moreover, sampled estuaries in higher latitudes are more exposed to human pressure but not especially covered by protection. Results highlight that, in many regions, efforts are needed to apply effective conservation measures within existing protected areas since little coverage is provided by protected areas with IUCN management categories I-IV.

Global conservation strategies should cover a network of locations/habitats (spatially nested within biogeographical regions) that protects several aspects of biodiversity - e.g. range rarity, low species resilience and high endemicity, taxonomic/functional diversity and sensitivity, as well as species vulnerability. Such strategies should consider that biogeographical region and ecosystem features regulate estuaries’ species richness^25^, composition^58^, functional traits^27^, as well as vulnerability and resilience (present study). Still, further research is needed for prioritizing particular sites, especially to account for effects of habitat complexity at local scales^59^ and on links between taxonomic and functional diversity.

## Materials and Methods

We compiled a comprehensive database for estuaries distributed worldwide (Fig. 1) on (a) fish assemblage composition in estuaries (Supplementary methods and Table S1), (b) ecosystem features of the sampled estuaries (Supplementary methods and Table S2) and (c) traits of the sampled fishes (Table 1). The database included 2434 taxa for 378 estuaries worldwide. Since estuaries are transition ecosystems we characterized human pressures as well as protection in and around each estuary (i.e. for marine, estuarine and freshwater ecosystems, but characterized separately per ecosystem).

### Fish assemblage data

We compiled a database of studies of fish assemblages in individual estuaries, aiming at a wide characterization of each estuary’s fish community (i.e. we excluded studies on dominant/selected taxa) and habitats (e.g. subtidal, tidal flats, creeks) but in some cases a complete characterization of habitats was not possible. To minimize sampling effects of different gear types we considered only active gears (e.g. trawl-, seine-, cast- nets, or trap-like gears such as enclosure nets/traps) and considered only surveys where total sampled area could be estimated so that it could be used to minimize sampling effort bias in subsequent analysis. Moreover, some estuaries are represented by more than one study in the database, and when possible, an estuary’s fish assemblage reported in a given study was treated separately by type of survey. Therefore, in this database, a sample consists of the fish assemblage sampled in a given estuary and survey (530 samples in 378 estuaries).

For each taxa we characterized ‘intrinsic extinction vulnerability to fishing^31^, coded using five categories (Table 1): low (<30%), low to moderate (30–40%), moderate to high (40–60%), high to very high (60–70%) and very high (>70%). This aggregate trait was parameterized with maximum body length, age at first maturity, von Bertalanffy growth parameter k, natural mortality rate, maximum age, geographic range, annual fecundity and strength of aggregation behaviour^31^. We also characterized ‘species productivity or resilience to fishing’^32^, i.e. minimum population doubling time, coded using four categories (Table 1): high (<1.4 yr.), medium (1.4–4.4 yr.), low (4.5–14 yr.) and very low (>14 yr.). This aggregate trait was determined through intrinsic rate of increase, von Bertalanffy k, fecundity, age at maturity and maximum age^32^. Finally, we also characterized the maximum body size of each species (small: <15 cm; medium: 15–50 cm; large: 50–100 cm; very large >100 cm). Traits were recorded using information available in FishBase (www.fishbase.org) and additional literature. Trait values were not available for <10% of the taxa (i.e. genus or families), which accounts for a mean of 5% and 7% per sample, for vulnerability and resilience, respectively. However, this percentage is consistent across continents, except it is higher in Africa, and lower in North America than Europe, due to the percentage of taxa resolved at species level (ANOVA and Tukey HSD, P < 0.05).

To evaluate the preponderance of the different trait categories in estuaries, we determined the “relative taxa richness” of each trait category per sample: i.e. the proportion of the taxa richness of a given trait category (e.g. high resilience) relative to the total observed taxa richness (i.e. richness = number of taxa). We used proportions to standardize among assemblages with different number of taxa resulting from different sampling effort. Moreover, we used taxa richness rather than abundance, since abundance data are available for less estuaries and we previously showed^27^ they both describe these assemblages in the same way.

### Biogeographical and environmental data

We determined a set of biogeographical and environmental variables for each estuary in the database (Supplementary methods and Table S2). Biogeographical location was characterized using continent and marine biogeographic realm^60^. Energy and productivity were described with latitude and temperature at the mouth of the estuary, and primary productivity of the adjoining marine and terrestrial ecosystems. Ecosystem size was described using area of the estuary and of the adjoining freshwater ecosystem (drainage basin), and continental shelf width was used as a proxy for the area of the adjoining marine coastal ecosystem. Hydrological connectivity of the estuary with the marine ecosystem was depicted with estuary type (open or temporarily-open), estuary mouth width and tidal range (macro-, meso- or microtidal). Finally, habitat suitability of the estuary was described in terms of salinity type (regular, regular-to-hyperhaline or hyperhaline).

### Human activities and pressures data

We characterized the potential level of exposure to human activity and pressure of the fish assemblage in and around each estuary (from marine, estuarine and freshwater ecosystems). We used data on drivers with acknowledged effect on ecosystem degradation, from reliable data sources and available at suitable coverage and resolution. Exposure to pressure in the marine ecosystem was measured with the index of cumulative human impact developed by Halpern and colleagues^12^. This index is based on 17 anthropogenic drivers of ecological change representing four main aspects: general, climate change, fishing and pollution. For each estuary in our database, we determined exposure to human pressure in the marine ecosystem (H_marine_) as the mean index in the coastal marine ecosystem (i.e. shallower than 200 m depth - continental shelf) within an influence radius defined by the size of that ecosystem (with 20, 40, 125, 440, 600 and 980 km radius respectively applied to the 25^th^, 50^th^, 75^th^, 90^th^, 95^th^ and 100 percentiles of continental shelf width in our database) (Supplementary Fig. S2).

To estimate exposure to human pressure in the estuarine ecosystem we used human population density around the estuaries. Human population density reflects a range of human driven impacts generated by multiple activities in and around estuaries (e.g. urban, industrial, rural, harbour, water use, resource exploitation) - for example, in previous studies Pearson correlation between human pressure and overall pressure was R^2^ = 0.63^61^ and R^2^ = 0.51^62^. For each site in our database, we quantified human pressure in the estuarine ecosystem (H_estuary_) as the mean population density (data for year 2000; http://sedac.ciesin.columbia.edu/data/set/gpw-v3-population-density ^63^) within an influence radius defined by estuary area (with 1, 5, 10, 30, 40, 265 km radius respectively applied to the 25^th^, 50^th^, 75^th^, 90^th^, 95^th^ and 100 percentiles of estuary area in our database) (Supplementary Fig. S2).

We evaluated exposure to human pressures in the freshwater ecosystem based on the cumulative incident threat index to river biodiversity developed by Vörösmarty and colleagues^18^. This index comprised 23 geospatial drivers under four themes: catchment disturbance, pollution, water resource development and biotic factors. For each estuary in the database, human pressure in the freshwater ecosystem (H_freshwater_) was quantified as the mean index within an influence radius covering the drainage basin area (with 15, 35, 85, 270, 315 and 1345 km radius respectively applied to the 25^th^, 50^th^, 75^th^, 90^th^, 95^th^ and 100 percentiles of drainage basin area in our database) (Supplementary Fig. S2).

### Protection data

To assess the potential protection of the fish assemblage in each estuary we determined the location of protected areas worldwide, by combining spatial data from the World Database on Protected Areas^64^ and MPAtlas^65^. Following an approach used in previous studies^10, 24^, two alternative selections of the database were done to address differences in terms of protection level: (a) PA_all_ - considering all protected areas; and (b) PA_I-IV_ - considering only protected areas classified with IUCN management categories I-IV (respectively strict nature reserve or wilderness area, national park, national monument or feature, habitat/species management area) which are protected areas that restrict human activities (e.g. fishing). For each estuary in the database, we quantified the extent of coverage by protected areas within an influence radius, in three ways, namely: PA_marine_ - i.e. protected areas in the marine ecosystem shallower than 200 m (continental shelf); PA_estuary_ - i.e. protected areas in and around the estuary; PA_freshwater_ - i.e. protected areas in and around the estuary but excluding the marine ecosystem. We used the radii used previously in the estimation of pressures. The extent of coverage by protected areas was calculated in area (km^2^) and in percentage (% of area that is protected within the influence radius). The lack of geospatial vector data for all estuaries and corresponding rivers/drainage basins precluded a more refined estimation of PA_estuary_ and PA_freshwater_. All pressure and protection data were compiled in ArcGIS for desktop version 10.4 (http://desktop.arcgis.com) using a Cylindrical Equal Area projection.

### Data analysis

Environmental, pressure and protection variables were logx + 1 transformed to reduce skewness and the effect of extreme observations^66, 67^. In addition, each pressure variable was normalized (scaled) so that 0 represents the lowest pressure and 1 the highest (from each value we took the minimum and divided by the range). Based on human pressures in the three ecosystems (H_marine_, H_estuary_, H_freshwater_) we also calculated: the mean of the three ecosystems (H_mean_), and the weighted mean of the three ecosystems (H_weighted-mean_) where, for each estuary, the weight of each ecosystem is given by the percentage of fish from that ecosystem in the estuarine assemblage (i.e. % marine fish for H_marine_, % resident fish for H_estuary_, % freshwater fish for H_freshwater_ and % diadromous fish for H_mean_).

We first examined the pairwise Pearson correlations between: all environmental variables, pressure variables and protection variables (R package stats); between all traits (relative taxa richness of trait categories); as well as between all traits and pressure/protection variables. To avoid effects of multicollinearity, several environmental variables were excluded from subsequent analyses, namely: latitude (with temperature), estuary mouth width and drainage basin area (with estuary area) (Table 3). We then used linear models (LM) to disentangle the relationship of fish traits (response variables) with all biogeographical and environmental variables (predictors). Additionally, since some estuaries have more than one sample in our database, we used linear mixed models (LMM) which were formulated similarly to the linear models but also included estuary as a random predictor. In both LM and LMM, sampling effort (i.e. total sampled area) was always included as a predictor to account for differences in effort between samples in our database. To attain robust estimates of the importance and parameter of each predictor, we implemented a multi model approach using: hierarchical partition of variation (R package relaimpo; only for LM) and multimodel inference (R package MuMIn; for both LM and LMM) which evaluate predictor importance respectively based on R^2^ and Akaike information criteria. Each trait category (e.g. proportion of taxa with low vulnerability) was modelled as a separate response variable, and for each trait category, we fitted two alternative models: with and without the biogeographical variables. As a note, categorical environmental variables were considered as continuous in correlation analysis and as ordered factors in linear- and linear mixed models (tidal regime: microtidal - 1, mesotidal - 2, macrotidal - 3; estuary type: temporarily open - 1, open - 2 salinity type: regular - 1, regular to hyperhaline - 2, hyperhaline - 3). A significance level of 0.05 was considered in all statistical analyses.

## Electronic supplementary material

Supplementary information 
