# Supplementary material for: Current limitations of global conservation to protect higher vulnerability and lower resilience fish species
Source: Sci Rep. 2017 Aug 9;7:7702. doi: 10.1038/s41598-017-06633-x (PMC5550462; doi:10.1038/s41598-017-06633-x)
Supplement: Supplementary file 1 — Supplementary information [file 41598_2017_6633_MOESM1_ESM.pdf]

Vasconcelos RP, Batista M, Henriques S. Current limitations of global conservation to protect higher vulnerability and lower resilience fish species.

1. Supplementary methods

1.1 CONSTRUCTION OF THE DATABASE

The database built included combined data on fish species assemblages in individual estuaries with global and local environmental variables in individual estuaries on a global scale. For construction of the database a set of criteria and rules were implemented to insure that a robust dataset was obtained. These pertained to three main aspects: (a) definition of estuary; (b) fish database - criteria for inclusion in the fish database, and data collected in each publication; (c) environmental database.

a) Definition of estuary

Potter and colleagues [1] reviewed the main concepts and definitions of estuaries that have been considered worldwide, from the work of Pritchard [2] and Day [3] to the revision by Elliott and McLusky [4], among others. They highlight that the majority of the definitions of estuaries have been based on the characteristics of estuaries in north temperate regions; that such definitions do not take into account features as periodic closure of their mouths and hypersaline conditions during dry periods, which characterise many estuaries in southern Africa and south-western Australia; and that there is ambiguity as to whether an estuary sensu stricto must be fed by a river. The definition of estuary considered in the present study was that developed by Potter and colleagues [1] to encompass the main characteristics of all estuaries in a global perspective: “An estuary is a partially enclosed coastal body of water that is either permanently or periodically open to the sea and which receives at least periodic discharge from a river(s), and thus, while its salinity is typically less than that of natural sea water and varies temporally and along its length, it can become hypersaline in regions when evaporative water loss is high and freshwater and tidal inputs are negligible”.

b) Fish community and sampling database

Criteria for inclusion in the fish database

Studies were searched in web of science and google scholar using a combination of the topics: (fish*) AND (assemblage* OR communit* OR fauna*) AND (estuar* OR lagoon* OR bay*).

Studies that met the following criteria were selected for inclusion in the fish database: (i) the system matched the considered definition of estuary; (ii) fish assemblages sampled in individual estuaries; (iii) the survey provides a wide characterization of the estuary’s fish assemblage - studies that focused on specific groups of species were excluded (e.g. excluded dominant species, residents, selected taxa); (iv) full species list with presence/absence or abundances - studies presenting only species number were excluded; (v) sampling clearly presented allowing to determine sampling effort, i.e. total area sampled - studies with unclear or unaccountable sampling methods were excluded (e.g. species checklists, fisheries landings). The search was generally directed at studies published after 1990, but time did not represent a criterion for inclusion in the database; and was completed before the end of 2013.

Data collected in each publication

From each study the following data were taken: (i) number of years, (ii) temporal gradient (annual, which specific seasons, isolated); temporal frequency (seasonal, bimonthly, monthly, isolated); (iii) salinity gradient (oligohaline 0.5-5, mesohaline 5-18, polyhaline-euhaline 18-40, hyperhaline >40 adapted from the work of Whitfield and colleagues [5]; (iv) type of fishing gears (e.g. beam trawl, beach seine); (v) number of fishing gear types used.

As for salinity gradient: in studies including sites in freshwater/limnetic reaches (salinity 0-0.5) and/or marine reaches outside the estuary mouth those sites were excluded, or when it was not possible to exclude those sites the study was excluded.

Regarding type of gear, whenever possible data was separated by type of gear; special care was taken to ensure that the same datasets were not duplicated (i.e. published on more than one paper). Fishing gear types used included chiefly: active (trawl-, seine-, beach seine-, purse seine-, cast- or hand/dip- nets) or trap-like gears (drop-, throw-, pop/lift- or enclosure-

nets/traps/samplers) - while passive gears were excluded (gill-, trammel-, fyke- or block/barrier/channel- nets, angling, hook and line fishing, weirs, water intake screens). Sampling effort (total sampled area in m^2) was determined aiming at obtaining a robust dataset and minimizing the effects of heterogeneity of sampling intensity. Gears such as plankton nets as well as visual census and electrofishing were excluded.

Species names were checked according to FishBase[6] (check names tool: match species names against FishBase) in order to standardize species lists into a single database. When studies listed some taxa at a higher taxonomic rank than species that taxa was considered.

c) Historic and environmental variables database

For each individual estuary included in the fish database a set of global and local environmental variables were compiled.

A first group of variables was estimated using Geographic Information Systems (ArcGIS 10.4 [7]) and publicly available global shapefiles: marine biogeographic realm (Spalding, et al. [8]); continent; latitude and longitude; continental shelf width (m, minimum distance to continental shelf limit considering the 150m bathymetry); estuary mouth width (m). This was also applied to Sea Surface Temperature (ºC) and chlorophyll *a* concentration ($mg.m^{-3}$) (both at closest point outside the estuary mouth, means between 2002 and 2009 at 5arcmin resolution; Tyberghein, et al. [9]) as well as to terrestrial net primary productivity ($gC.m^{-2}.day^{-1}$; at closest point to the estuary mouth at 60 arcmin resolution; Foley, et al. [10] and Kucharik, et al. [11]).

A second group of variables was quantified based on published data from multiple sources (papers, theses, reports, official institutional or governmental websites): estuary type (open, permanently open); drainage basin area (km^2); estuary area (km^2); tidal regime (microtidal <2m, mesotidal 2-4m, macrotidal >4m); annual mean river flow (m^3 s^{-1}); and estuary salinity type [hyperhaline (typically with areas >40), regular to hyperhaline (occasionally >40), regular (<40)].

Additional local variables were initially compiled but they were removed from the database as they could not be determined for all estuaries: intertidal area (km^2), maximum depth (m), average depth (m), area covered by different habitats (km^2) - for habitat availability; tidal range (m) - for connectivity with adjacent marine ecosystem; dissolved oxygen ($mg.L^{-1}$), turbidity (NTU) and pH - for habitat suitability; and clorophyll a concentration ($mg.m^{-3}$) - for estuary primary productivity.

When it was not possible to estimate a minimum set of environmental variables for a given estuary it was excluded from the database and analysis.

2. Supplementary tables

Table S1. Estuaries and references of the studies used to construct the worldwide fish assemblages database. Abbreviations of marine biogeographic realms are: TSAf - Temperate Southern Africa, TNA - Temperate North Atlantic, TNP - Temperate North Pacific, TSAm - Temperate South America, TAu - Temperate Australasia, TAt - Tropical Atlantic, TEP - Tropical Eastern Pacific, WIP - Western Indo Pacific, CIP - Central Indo Pacific, A- Artic; and abbreviations of continents are: AFR - Africa, EUR - Europe, NAM - North America, SAM - South America, ASI - Asia, OCE- Oceania. Each line is a sample in the database, i.e. the fish assemblage sampled in a given estuary and survey (total number of samples 530, total number of estuaries 378); some samples correspond to the same estuary and reference but result from separate surveys (e.g. different sampling gears) with explicitly different assemblages.

Estuary	Country	Continent	Realm	Reference
Kariega	South Africa	AFR	TSAf	Wasserman and Strydom [12]
Kariega	South Africa	AFR	TSAf	Bailey and James [13]
Kariega	South Africa	AFR	TSAf	Whitfield and Paterson [14]
Great Fish	South Africa	AFR	TSAf	Wasserman and Strydom [12]
Kowie	South Africa	AFR	TSAf	Wasserman and Strydom [12]
Sundays	South Africa	AFR	TSAf	Wasserman and Strydom [12]
Kasuka	South Africa	AFR	TSAf	Lukey, Booth and Froneman [15]
East-Kleinemonde	South Africa	AFR	TSAf	James, Whitfield and Cowley [16]
East-Kleinemonde	South Africa	AFR	TSAf	James, Whitfield and Cowley [16]
Mfolozi/Msunduzi	South Africa	AFR	TSAf	Vivier, Cyrus, Owen and Jerling [17]
Thukela	South Africa	AFR	TSAf	Withfield and Harrison [18]
Sine Saloum	Senegal	AFR	TAt	Simier, Blanc, Aliaume, Diouf and Albaret [19]
Sine Saloum	Senegal	AFR	TAt	Faye, et al. [20]
Gazi Bay	Kenya	AFR	WIP	Huxham, Kimani and Augley [21]
Gazi Bay	Kenya	AFR	WIP	Crona and Rönnbäck [22]
Gazi Bay	Kenya	AFR	WIP	de Troch, Mees, Papadopoulos and Wakwabi [23]
Gazi Bay	Kenya	AFR	WIP	Huxham, Kimani and Augley [24]
Ebrie lagoon	Ivory coast	AFR	TAt	Ecoutin, Richard, Simier and Albaret [25]
Mhlanga	South Africa	AFR	TSAf	Harrison [26]
Mhlanga	South Africa	AFR	TSAf	Harrison [26]
Mhlanga	South Africa	AFR	TSAf	Harrison [26]
Zotsha	South Africa	AFR	TSAf	Harrison [26]
Zotsha	South Africa	AFR	TSAf	Harrison [26]
Zotsha	South Africa	AFR	TSAf	Harrison [26]
Shatt Al-Arab river	Iraq	AFR	WIP	Mohamed, Resen and Taher [27]
Mngazi	South Africa	AFR	TSAf	Grant [28]
Mngazana	South Africa	AFR	TSAf	Grant [28]
Sundays	South Africa	AFR	TSAf	Beckley [29]
Olifants	South Africa	AFR	TSAf	Harrison [30]
Berg	South Africa	AFR	TSAf	Harrison [30]
Diep	South Africa	AFR	TSAf	Harrison [30]
Wildevoel	South Africa	AFR	TSAf	Harrison [30]
Krom	South Africa	AFR	TSAf	Harrison [30]
Sand	South Africa	AFR	TSAf	Harrison [30]
Palmiet	South Africa	AFR	TSAf	Harrison [30]
Uilkraals	South Africa	AFR	TSAf	Harrison [30]
Heuningnes	South Africa	AFR	TSAf	Harrison [30]
Bree	South Africa	AFR	TSAf	Harrison [30]
Duiwenhoks	South Africa	AFR	TSAf	Harrison [30]

Gourits	South Africa	AFR	TSAf	Harrison [30]
Blinde	South Africa	AFR	TSAf	Harrison [30]
Hartenbos	South Africa	AFR	TSAf	Harrison [30]
Keurbooms	South Africa	AFR	TSAf	Harrison [30]
Groot	South Africa	AFR	TSAf	Harrison [30]
Tsitsikamma	South Africa	AFR	TSAf	Harrison [30]
Kromme	South Africa	AFR	TSAf	Harrison [30]
Seekoei	South Africa	AFR	TSAf	Harrison [30]
Kabeljous	South Africa	AFR	TSAf	Harrison [30]
Gamtoos	South Africa	AFR	TSAf	Harrison [30]
Van Stadens	South Africa	AFR	TSAf	Harrison [30]
Swartkops	South Africa	AFR	TSAf	Harrison [30]
Sundays	South Africa	AFR	TSAf	Harrison [30]
Boknes	South Africa	AFR	TSAf	Harrison [30]
Bushmans	South Africa	AFR	TSAf	Harrison [30]
Kariega	South Africa	AFR	TSAf	Harrison [30]
Kasuka	South Africa	AFR	TSAf	Harrison [30]
Kowie	South Africa	AFR	TSAf	Harrison [30]
Riet	South Africa	AFR	TSAf	Harrison [30]
West-Kleinemonde	South Africa	AFR	TSAf	Harrison [30]
East-Kleinemonde	South Africa	AFR	TSAf	Harrison [30]
Great Fish	South Africa	AFR	TSAf	Harrison [30]
Old Woman	South Africa	AFR	TSAf	Harrison [30]
Mpekweni	South Africa	AFR	TSAf	Harrison [30]
Mtati	South Africa	AFR	TSAf	Harrison [30]
Mgwalana	South Africa	AFR	TSAf	Harrison [30]
Bira	South Africa	AFR	TSAf	Harrison [30]
Gqutywa	South Africa	AFR	TSAf	Harrison [30]
Mtana	South Africa	AFR	TSAf	Harrison [30]
Keiskamma	South Africa	AFR	TSAf	Harrison [30]
Nqinisa	South Africa	AFR	TSAf	Harrison [30]
Kiwane	South Africa	AFR	TSAf	Harrison [30]
Ross Creek	South Africa	AFR	TSAf	Harrison [30]
Ncera	South Africa	AFR	TSAf	Harrison [30]
Mlele	South Africa	AFR	TSAf	Harrison [30]
Mcantsi	South Africa	AFR	TSAf	Harrison [30]
Gxulu	South Africa	AFR	TSAf	Harrison [30]
Goda	South Africa	AFR	TSAf	Harrison [30]
Hickmans	South Africa	AFR	TSAf	Harrison [30]
Buffalo	South Africa	AFR	TSAf	Harrison [30]
Nahoon	South Africa	AFR	TSAf	Harrison [30]
Qinira	South Africa	AFR	TSAf	Harrison [30]
Gqunube	South Africa	AFR	TSAf	Harrison [30]
Kwelera	South Africa	AFR	TSAf	Harrison [30]
Cintsa	South Africa	AFR	TSAf	Harrison [30]
Cefane	South Africa	AFR	TSAf	Harrison [30]
Kwenxura	South Africa	AFR	TSAf	Harrison [30]
Nyara	South Africa	AFR	TSAf	Harrison [30]

Haga-Haga	South Africa	AFR	TSAf	Harrison [30]
Morgan	South Africa	AFR	TSAf	Harrison [30]
Great-Kei	South Africa	AFR	TSAf	Harrison [30]
Gxara	South Africa	AFR	TSAf	Harrison [30]
Ngogwane	South Africa	AFR	TSAf	Harrison [30]
Qolora	South Africa	AFR	TSAf	Harrison [30]
Kobonqaba	South Africa	AFR	TSAf	Harrison [30]
Ngqusi/Inxaxo	South Africa	AFR	TSAf	Harrison [30]
Cebe	South Africa	AFR	TSAf	Harrison [30]
Zalu	South Africa	AFR	TSAf	Harrison [30]
Ngqwara	South Africa	AFR	TSAf	Harrison [30]
Qora	South Africa	AFR	TSAf	Harrison [30]
Shixini	South Africa	AFR	TSAf	Harrison [30]
Mbashe	South Africa	AFR	TSAf	Harrison [30]
Xora	South Africa	AFR	TSAf	Harrison [30]
Mtata	South Africa	AFR	TSAf	Harrison [30]
Mdumbi	South Africa	AFR	TSAf	Harrison [30]
Mngazana	South Africa	AFR	TSAf	Harrison [30]
Mngazi	South Africa	AFR	TSAf	Harrison [30]
Mntafufu	South Africa	AFR	TSAf	Harrison [30]
Msikaba	South Africa	AFR	TSAf	Harrison [30]
Mtentu	South Africa	AFR	TSAf	Harrison [30]
Mzamba	South Africa	AFR	TSAf	Harrison [30]
Mtentwana	South Africa	AFR	TSAf	Harrison [30]
Kandandlovu	South Africa	AFR	TSAf	Harrison [30]
Mpenjati	South Africa	AFR	TSAf	Harrison [30]
Umhlangankulu	South Africa	AFR	TSAf	Harrison [30]
Kaba	South Africa	AFR	TSAf	Harrison [30]
Mbizana	South Africa	AFR	TSAf	Harrison [30]
Bilanhlolo	South Africa	AFR	TSAf	Harrison [30]
Mhlangeni	South Africa	AFR	TSAf	Harrison [30]
Mzimkulu	South Africa	AFR	TSAf	Harrison [30]
Mtentweni	South Africa	AFR	TSAf	Harrison [30]
Mhlangamkulu	South Africa	AFR	TSAf	Harrison [30]
Intshambili	South Africa	AFR	TSAf	Harrison [30]
Fafa	South Africa	AFR	TSAf	Harrison [30]
Sezela	South Africa	AFR	TSAf	Harrison [30]
Mpambanyoni	South Africa	AFR	TSAf	Harrison [30]
Mahlongwa	South Africa	AFR	TSAf	Harrison [30]
Mkomazi	South Africa	AFR	TSAf	Harrison [30]
Little Manzimtoti	South Africa	AFR	TSAf	Harrison [30]
Manzimtoti	South Africa	AFR	TSAf	Harrison [30]
Mhlanga	South Africa	AFR	TSAf	Harrison [30]
Mdloti	South Africa	AFR	TSAf	Harrison [30]
Mdlotane	South Africa	AFR	TSAf	Harrison [30]
Zinkwasi	South Africa	AFR	TSAf	Harrison [30]
Matigulu/Nyoni	South Africa	AFR	TSAf	Harrison [30]
Siyai	South Africa	AFR	TSAf	Harrison [30]

Mlalazi	South Africa	AFR	TSAf	Harrison [30]
Bira	South Africa	AFR	TSAf	Vorwerk [31]
East-Kleinemonde	South Africa	AFR	TSAf	Vorwerk [31]
Gqutywa	South Africa	AFR	TSAf	Vorwerk [31]
Great fish	South Africa	AFR	TSAf	Vorwerk [31]
Keiskamma	South Africa	AFR	TSAf	Vorwerk [31]
Kleine Palmiet estuary	South Africa	AFR	TSAf	Vorwerk [31]
Mgwalana	South Africa	AFR	TSAf	Vorwerk [31]
Mpekweni	South Africa	AFR	TSAf	Vorwerk [31]
Mtati	South Africa	AFR	TSAf	Vorwerk [31]
Ngculura	South Africa	AFR	TSAf	Vorwerk [31]
Bira	South Africa	AFR	TSAf	Vorwerk [31]
East-Kleinemonde	South Africa	AFR	TSAf	Vorwerk [31]
Gqutywa	South Africa	AFR	TSAf	Vorwerk [31]
Great fish	South Africa	AFR	TSAf	Vorwerk [31]
Kleine Palmiet estuary	South Africa	AFR	TSAf	Vorwerk [31]
Mgwalana	South Africa	AFR	TSAf	Vorwerk [31]
Mpekweni	South Africa	AFR	TSAf	Vorwerk [31]
Mtati	South Africa	AFR	TSAf	Vorwerk [31]
Yangtze/Changjiang	China	ASI	TNP	Quan, Shi and Chen [32]
Sikao Creek	Thailand	ASI	WIP	Tongnunui, et al. [33]
Sikao Creek	Thailand	ASI	WIP	Tongnunui, et al. [33]
Pak Phanang Bay	Thailand	ASI	CIP	Shinnaka, et al. [34]
Gesashi	Japan	ASI	CIP	Tachihara, et al. [35]
Shimajiri	Japan	ASI	CIP	Tachihara, et al. [35]
Nagura	Japan	ASI	CIP	Tachihara, et al. [35]
Urauchi	Japan	ASI	CIP	Tachihara, et al. [35]
Bakkhali	Bangladesh	ASI	WIP	Rashed-Un-Nabi, Al-Mamun, Ullah and Mustafa [36]
Pattani Bay	Thailand	ASI	CIP	Hajisamae, Yeesin and Chaimongkol [37]
Tokyo Bay	Japan	ASI	TNP	Kanou, Sano and Kohno [38]
Negombo	Sri Lanka	ASI	WIP	Pinto and Punchihewa [39]
Dongzhaigang Bay	China	ASI	CIP	Wang, Huang, Shi and Wang [40]
Tokyo Bay	Japan	ASI	TNP	Hermosilla, Tamura, Moteki and Kohno [42]
Illawarra Lake	Australia	OCE	TAu	Griffiths [43]
Werri Lagoon	Australia	OCE	TAu	Griffiths [43]
Shellharbour Lagoon	Australia	OCE	TAu	Griffiths [43]
Shellharbour Lagoon	Australia	OCE	TAu	Griffiths [44]
Swan-Canning	Australia	OCE	TAu	Hoeksema and Potter [45]
Swan-Canning	Australia	OCE	TAu	Kanandjembo, Potter and Platell [46]
Swan-Canning	Australia	OCE	TAu	Loneragan, Potter and Lenanton [47]
Blackwood	Australia	OCE	TAu	Valesini, Potter, Platell and Hyndes [48]
Spencer Gulf	Australia	OCE	TAu	Akin, Buhan, Winemiller and Yilmaz [49]
Embley	Australia	OCE	CIP	Vance, et al. [50]
Barker Inlet-Port River	Australia	OCE	TAu	Bloomfield and Gillanders [51]
Barker Inlet-Port River	Australia	OCE	TAu	Bloomfield and Gillanders [51]
Barker Inlet-Port River	Australia	OCE	TAu	Jackson and Jones [52]
Barker Inlet-Port River	Australia	OCE	TAu	Jones, Baker, Edyvane and Wright [53]
Tweed	Australia	OCE	TAu	Gray, McElligott and Chick [54]

Cudgera Creek	Australia	OCE	TAu	Gray, McElligott and Chick [54]
Brunswick	Australia	OCE	TAu	Gray, McElligott and Chick [54]
Richmond	Australia	OCE	TAu	Gray, McElligott and Chick [54]
Clarence	Australia	OCE	TAu	Gray, McElligott and Chick [54]
Sandon	Australia	OCE	TAu	Gray, McElligott and Chick [54]
Wooli Wooli	Australia	OCE	TAu	Gray, McElligott and Chick [54]
Corindi	Australia	OCE	TAu	Gray, McElligott and Chick [54]
Clarence	Australia	OCE	TAu	Rotherham, Broadhurst, Gray and Johnson [55]
Clarence	Australia	OCE	TAu	West [56]
Clarence	Australia	OCE	TAu	West [56]
Tuggerah Lake	Australia	OCE	TAu	Rotherham, Gray, Johnson and Lokys [57]
Werri Lagoon	Australia	OCE	TAu	Griffiths and West [58]
Fairy Creek	Australia	OCE	TAu	Griffiths and West [58]
Bellambi Lagoon	Australia	OCE	TAu	Griffiths and West [58]
Broke Inlet	Australia	OCE	TAu	Hoeksema, Chuwen and Potter [59]
Irwin Inlet	Australia	OCE	TAu	Hoeksema, Chuwen and Potter [59]
Oyster Harbour	Australia	OCE	TAu	Hoeksema, Chuwen and Potter [59]
Wellstead	Australia	OCE	TAu	Hoeksema, Chuwen and Potter [59]
Wilson Inlet	Australia	OCE	TAu	Hoeksema, Chuwen and Potter [59]
Wilson Inlet	Australia	OCE	TAu	Humphries, Potter and Loneragan [60]
Wilson Inlet	Australia	OCE	TAu	Potter, Hyndes and Baronie [61]
Nornalup-Walpole	Australia	OCE	TAu	Potter and Hyndes [62]
Peel-Harvey	Australia	OCE	TAu	Potter, Loneragan, Lenanton, Chrystal and Grant [63]
Peel-Harvey	Australia	OCE	TAu	Potter, Loneragan, Lenanton, Chrystal and Grant [63]
Peel-Harvey	Australia	OCE	TAu	Young and Potter [64]
Manukau Harbour	New Zealand	OCE	TAu	Morrison, Francis, Hartill and Parkinson [65]
Manukau Harbour	New Zealand	OCE	TAu	Morrison, Francis, Hartill and Parkinson [65]
Moreton Bay	Australia	OCE	TAu	Morton [66]
Moreton Bay	Australia	OCE	TAu	Morton [66]
Moreton Bay	Australia	OCE	TAu	Quinn [67]
Botany Bay	Australia	OCE	TAu	Rotherham and West [68]
Port Hacking	Australia	OCE	TAu	Rotherham and West [68]
Sussex Inlet-St Georges Basin	Australia	OCE	TAu	Rotherham and West [68]
Alligator Creek	Australia	OCE	CIP	Sheaves and Johnston [69]
Armstrong Creek	Australia	OCE	CIP	Sheaves and Johnston [69]
Barratta Creek	Australia	OCE	CIP	Sheaves and Johnston [69]
Constant Creek	Australia	OCE	CIP	Sheaves and Johnston [69]
Crocodile Creek	Australia	OCE	CIP	Sheaves and Johnston [69]
Deluge Inlet	Australia	OCE	CIP	Sheaves and Johnston [69]
Haughton River	Australia	OCE	CIP	Sheaves and Johnston [69]
Hull River	Australia	OCE	CIP	Sheaves and Johnston [69]
Meunga Creek	Australia	OCE	CIP	Sheaves and Johnston [69]
Morris Creek	Australia	OCE	CIP	Sheaves and Johnston [69]
Mossman River	Australia	OCE	CIP	Sheaves and Johnston [69]
Murray Creek	Australia	OCE	CIP	Sheaves and Johnston [69]
Murray River	Australia	OCE	CIP	Sheaves and Johnston [69]
Neames Inlet	Australia	OCE	CIP	Sheaves and Johnston [69]
Packer Creek	Australia	OCE	CIP	Sheaves and Johnston [69]

Rocky Ponds Creek	Australia	OCE	CIP	Sheaves and Johnston [69]
Ross River	Australia	OCE	CIP	Sheaves and Johnston [69]
Saltwater Creek	Australia	OCE	CIP	Sheaves and Johnston [69]
Victor Creek	Australia	OCE	CIP	Sheaves and Johnston [69]
Waterfall Creek	Australia	OCE	CIP	Sheaves and Johnston [69]
Yellow Gin Creek	Australia	OCE	CIP	Sheaves and Johnston [69]
Moore	Australia	OCE	TAu	Young, Potter, Hyndes and de Lestang [70]
Leschenault	Australia	OCE	TAu	Potter, Tiivel, Valesini and Hyndes [71]
Trinity	Australia	OCE	CIP	Blaber [72]
Trinity	Australia	OCE	CIP	Blaber [72]
Gironde estuary	France	EUR	TNA	Pasquaud, et al. [73]
Gironde estuary	France	EUR	TNA	Pasquaud, et al. [73]
Mira estuary	Portugal	EUR	TNA	Cardoso, et al. [74]
Odeceixe estuary	Portugal	EUR	TNA	Cardoso, et al. [74]
Alzejur estuary	Portugal	EUR	TNA	Cardoso, et al. [74]
bensafrim estuary	Portugal	EUR	TNA	Cardoso, et al. [74]
Gilao estuary	Portugal	EUR	TNA	Cardoso, et al. [74]
Minho estuary	Portugal	EUR	TNA	Costa-Dias, Freitas, Sousa and Antunes [75]
Minho estuary	Portugal	EUR	TNA	França, Costa and Cabral [76]
Douro estuary	Portugal	EUR	TNA	França, Costa and Cabral [76]
Ria de Aveiro	Portugal	EUR	TNA	França, Costa and Cabral [76]
Mondego estuary	Portugal	EUR	TNA	França, Costa and Cabral [76]
Tejo estuary	Portugal	EUR	TNA	França, Costa and Cabral [76]
Sado estuary	Portugal	EUR	TNA	França, Costa and Cabral [76]
Mira estuary	Portugal	EUR	TNA	França, Costa and Cabral [76]
Ria Formosa	Portugal	EUR	TNA	França, Costa and Cabral [76]
Guadiana estuary	Portugal	EUR	TNA	França, Costa and Cabral [76]
Novigrad sea	Croatia	EUR	TNA	Matič-Skoko, Peharda, Pallaoro, Cukrov and Baždarič [77]
Tejo estuary	Portugal	EUR	TNA	Thiel, Cabral and Costa [78]
Canche estuary	France	EUR	TNA	Selleslagh and Amara [79]
Ria Formosa	Portugal	EUR	TNA	Ribeiro, et al. [80]
Guadiana estuary	Portugal	EUR	TNA	Veiga, Vieira, Bexiga, Sá and Erzini [81]
Obidos lagoon	Portugal	EUR	TNA	Gordo and Cabral [82]
Venice lagoon	Italy	EUR	TNA	Franco, Malavasi, Zucchetta, Franzoi and Torricelli [83]
Ria de Aveiro	Portugal	EUR	TNA	Pombo, Rebelo and Elliott [84]
Humber estuary	UK	EUR	TNA	Marshall and Elliott [85]
Tyne estuary	UK	EUR	TNA	Pomfret, Turner and Phillips [86]
Mondego estuary	Portugal	EUR	TNA	Martinho, et al. [87]
Thames estuary	UK (England)	EUR	TNA	Araújo, Bailey and Williams [88]
Tejo estuary	Portugal	EUR	TNA	Costa and Bruxelas [89]
Forth estuary	UK (Scotland/England)	EUR	TNA	Elliott, O´Reilly and Taylor [90]
Scheldt estuary	Belgium/Netherlands	EUR	TNA	Hamerlynck, Hostens, Arellano, Mees and Van Damme [91]
Scheldt estuary	Belgium/Netherlands	EUR	TNA	Hamerlynck, Hostens, Arellano, Mees and Van Damme [91]
Scheldt estuary	Belgium/Netherlands	EUR	TNA	Hostens [92]
Porto-Lagos lagoon	Greece	EUR	TNA	Koutrakis, Tsikliras and Sinis [93]
Salse-Leucate Lagoon	France	EUR	TNA	Mouillot, Dumay and Tomasini [94]
Saint-Nazaire Lagoon	France	EUR	TNA	Mouillot, Dumay and Tomasini [94]
Ria Formosa	Portugal	EUR	TNA	Ribeiro, et al. [95]

Authie estuary	France	EUR	TNA	Selleslagh, et al. [96]
Somme estuary	France	EUR	TNA	Selleslagh, et al. [96]
Barbadun estuary	Spain (Basque Country)	EUR	TNA	Uriarte and Borja [97]
Nervion estuary	Spain (Basque Country)	EUR	TNA	Uriarte and Borja [97]
Butroe estuary	Spain (Basque Country)	EUR	TNA	Uriarte and Borja [97]
Oka estuary	Spain (Basque Country)	EUR	TNA	Uriarte and Borja [97]
Lea estuary	Spain (Basque Country)	EUR	TNA	Uriarte and Borja [97]
Artibai estuary	Spain (Basque Country)	EUR	TNA	Uriarte and Borja [97]
Deba estuary	Spain (Basque Country)	EUR	TNA	Uriarte and Borja [97]
Urola estuary	Spain (Basque Country)	EUR	TNA	Uriarte and Borja [97]
Oria estuary	Spain (Basque Country)	EUR	TNA	Uriarte and Borja [97]
Urumea estuary	Spain (Basque Country)	EUR	TNA	Uriarte and Borja [97]
Oiartzun estuary	Spain (Basque Country)	EUR	TNA	Uriarte and Borja [97]
Bidasoa estuary	Spain (Basque Country)	EUR	TNA	Uriarte and Borja [97]
Mar Menor lagoon	Spain	EUR	TNA	Verdiell-Cubedo et al[98]
Po river delta	Italy	EUR	TNA	Franzoi, Trisolini, Carrieri and Rossi [100]
Vistula Lagoon	Poland	EUR	TNA	Wilkońska and Kapusta [101]
Guadiana estuary	Portugal	EUR	TNA	Nicolas, et al. [102]
Mira estuary	Portugal	EUR	TNA	Nicolas, et al. [102]
Sado estuary	Portugal	EUR	TNA	Nicolas, et al. [102]
Tejo estuary	Portugal	EUR	TNA	Nicolas, et al. [102]
Douro estuary	Portugal	EUR	TNA	Nicolas, et al. [102]
barbadun estuary	Spain (Basque Country)	EUR	TNA	Nicolas, et al. [102]
Nervion estuary	Spain (Basque Country)	EUR	TNA	Nicolas, et al. [102]
Butroe estuary	Spain (Basque Country)	EUR	TNA	Nicolas, et al. [102]
Artibai estuary	Spain (Basque Country)	EUR	TNA	Nicolas, et al. [102]
Urola estuary	Spain (Basque Country)	EUR	TNA	Nicolas, et al. [102]
Oria estuary	Spain (Basque Country)	EUR	TNA	Nicolas, et al. [102]
Bidasoa estuary	Spain (Basque Country)	EUR	TNA	Nicolas, et al. [102]
Adour estuary	France	EUR	TNA	Nicolas, et al. [102]
Gironde estuary	France	EUR	TNA	Nicolas, et al. [102]
Seudre estuary	France	EUR	TNA	Nicolas, et al. [102]
Charente estuary	France	EUR	TNA	Nicolas, et al. [102]
Sevre Niortaise estuary	France	EUR	TNA	Nicolas, et al. [102]
Loire estuary	France	EUR	TNA	Nicolas, et al. [102]
Vilaine estuary	France	EUR	TNA	Nicolas, et al. [102]
Goyen estuary	France	EUR	TNA	Nicolas, et al. [102]
Morlaix estuary	France	EUR	TNA	Nicolas, et al. [102]
Trieux estuary	France	EUR	TNA	Nicolas, et al. [102]
Mont Saint Michel bay	France	EUR	TNA	Nicolas, et al. [102]
Veys bay	France	EUR	TNA	Nicolas, et al. [102]
Orne estuary	France	EUR	TNA	Nicolas, et al. [102]
Seine estuary	France	EUR	TNA	Nicolas, et al. [102]
Somme estuary	France	EUR	TNA	Nicolas, et al. [102]
Authie estuary	France	EUR	TNA	Nicolas, et al. [102]
Canche estuary	France	EUR	TNA	Nicolas, et al. [102]
Cromarty estuary	UK (Scotland)	EUR	TNA	Nicolas, et al. [102]
Moray Beauly estuary	UK (Scotland)	EUR	TNA	Nicolas, et al. [102]

Tay estuary	UK (Scotland)	EUR	TNA	Nicolas, et al. [102]
Forth estuary	UK (Scotland/England)	EUR	TNA	Nicolas, et al. [102]
Tweed estuary	UK (England)	EUR	TNA	Nicolas, et al. [102]
Tyne estuary	UK (England)	EUR	TNA	Nicolas, et al. [102]
Wear estuary	UK (England)	EUR	TNA	Nicolas, et al. [102]
Tees estuary	UK (England)	EUR	TNA	Nicolas, et al. [102]
Humber estuary	UK (England)	EUR	TNA	Nicolas, et al. [102]
Ore estuary	UK (England)	EUR	TNA	Nicolas, et al. [102]
Orwell estuary	UK (England)	EUR	TNA	Nicolas, et al. [102]
Blackwater and Coln estuaries	UK (England)	EUR	TNA	Nicolas, et al. [102]
Crouch estuary	UK (England)	EUR	TNA	Nicolas, et al. [102]
Thames estuary	UK (England)	EUR	TNA	Nicolas, et al. [102]
Swale estuary	UK (England)	EUR	TNA	Nicolas, et al. [102]
Cuckmere estuary	UK (England)	EUR	TNA	Nicolas, et al. [102]
Adur estuary	UK (England)	EUR	TNA	Nicolas, et al. [102]
Arun estuary	UK (England)	EUR	TNA	Nicolas, et al. [102]
Exe estuary	UK (England)	EUR	TNA	Nicolas, et al. [102]
Dart estuary	UK (England)	EUR	TNA	Nicolas, et al. [102]
Fal estuary	UK (England)	EUR	TNA	Nicolas, et al. [102]
Severn estuary	UK (England)	EUR	TNA	Nicolas, et al. [102]
Loughor estuary	UK (Wales)	EUR	TNA	Nicolas, et al. [102]
Tywi estuary	UK (Wales)	EUR	TNA	Nicolas, et al. [102]
Cleddau estuary	UK (Wales)	EUR	TNA	Nicolas, et al. [102]
Dovey estuary	UK (Wales)	EUR	TNA	Nicolas, et al. [102]
Mawddach estuary	UK (Wales)	EUR	TNA	Nicolas, et al. [102]
Glaslyn estuary	UK (Wales)	EUR	TNA	Nicolas, et al. [102]
Conwy estuary	UK (Wales)	EUR	TNA	Nicolas, et al. [102]
Dee estuary	UK (Wales)	EUR	TNA	Nicolas, et al. [102]
Mersey estuary	UK (England)	EUR	TNA	Nicolas, et al. [102]
Ribble estuary	UK (England)	EUR	TNA	Nicolas, et al. [102]
Wyre estuary	UK (England)	EUR	TNA	Nicolas, et al. [102]
Lune estuary	UK (England)	EUR	TNA	Nicolas, et al. [102]
Kent estuary	UK (England)	EUR	TNA	Nicolas, et al. [102]
Leven estuary	UK (England)	EUR	TNA	Nicolas, et al. [102]
Lagan estuary	UK (Northern Ireland)	EUR	TNA	Nicolas, et al. [102]
Newry estuary	UK (Northern Ireland)	EUR	TNA	Nicolas, et al. [102]
Dundalk bay	Ireland	EUR	TNA	Nicolas, et al. [102]
Boyne estuary	Ireland	EUR	TNA	Nicolas, et al. [102]
Rogerstown estuary	Ireland	EUR	TNA	Nicolas, et al. [102]
Liffey estuary	Ireland	EUR	TNA	Nicolas, et al. [102]
Suir estuary	Ireland	EUR	TNA	Nicolas, et al. [102]
Bandon estuary	Ireland	EUR	TNA	Nicolas, et al. [102]
Newport bay (EUR)	Ireland	EUR	TNA	Nicolas, et al. [102]
Tullaghan bay	Ireland	EUR	TNA	Nicolas, et al. [102]
Sruwaddacon bay	Ireland	EUR	TNA	Nicolas, et al. [102]
Moy estuary	Ireland	EUR	TNA	Nicolas, et al. [102]
Gweebarra estuary	Ireland	EUR	TNA	Nicolas, et al. [102]
Faughan estuary	UK (Northern Ireland)	EUR	TNA	Nicolas, et al. [102]

Roe estuary	UK (Northern Ireland)	EUR	TNA	Nicolas, et al. [102]
bann estuary	UK (Northern Ireland)	EUR	TNA	Nicolas, et al. [102]
Fogliano lagoon	Italy	EUR	TNA	Mariani [103]
Caprolace lagoon	Italy	EUR	TNA	Mariani [103]
Lesina lagoon	Italy	EUR	TNA	Hadderingh and Jager [104]
Fogliano lagoon	Italy	EUR	TNA	Hadderingh and Jager [104]
Caprolace lagoon	Italy	EUR	TNA	Hadderingh and Jager [104]
Pantan estuary	Croatia	EUR	TNA	Hadderingh and Jager [104]
Oslo fjord	Norway	EUR	TNA	Nash [105]
Yaquina Bay	USA	NAM	TNP	De Ben, Clothier, Ditsworth and Baumgartner [106]
Elkhorn Slough	USA	NAM	TNP	Nybakken, Cailliet and Broenkow [107]
Elkhorn Slough	USA	NAM	TNP	Nybakken, Cailliet and Broenkow [107]
Lake Pontchartrai	USA	NAM	TNA	Duffy and Baltz [108]
Matagorda Bay	USA	NAM	TNA	Gelwick, Akin, Arrington and Winemiller [110]
Great Bay (NJ)	USA	NAM	TNA	Hagan and Able [111]
Chesapeake Bay	USA	NAM	TNA	Jung and Houde [112]
Casco Bay	USA	NAM	TNA	Lazzari, Sherman and Kanwit [113]
Muscongus Bay	USA	NAM	TNA	Lazzari, Sherman and Kanwit [113]
Weskeag River	USA	NAM	TNA	Lazzari, Sherman and Kanwit [113]
Chesapeake Bay	USA	NAM	TNA	Vieira [114]
Tampa Bay	USA	NAM	TAt	Greenwood, Matheson Jr., McMichael Jr. and MacDonald [115]
Saco River	USA	NAM	TNA	Furey and Sulikowski [116]
Chesapeake Bay	USA	NAM	TNA	Wingate and Secor [117]
Delaware Bay	USA	NAM	TNA	Able, Nemerson, Bush and Light [118]
Bellevue	Canada	NAM	A	Methven, Haedrich and Rose [119]
Chesapeake Bay	USA	NAM	TNA	Bilkovic, Hershner and Angstadt [120]
Matagorda Bay	USA	NAM	TNA	Akin, Winemiller and Gelwick [121]
Barataria Bay - Caminada Bay	USA	NAM	TNA	Baltz, Rakocinski and Fleeger [122]
Charlotte Harbour	USA	NAM	TAt	Fraser [123]
Atchafalaya - Vermilion Bays	USA	NAM	TNA	Baltz and Jones [124]
Galveston Bay	USA	NAM	TNA	Rozas, Minello, Zimmerman and Caldwell [125]
San Francisco Bay	USA	NAM	TNP	Moyle, Daniels, Herbold and Baltz [126]
Galveston Bay	USA	NAM	TNA	Stunz, Minello and Rozas [127]
Great Bay (NJ)	USA	NAM	TNA	Martino and Able [128]
Hudson River	USA	NAM	TNA	Hurst, McKown and Conover [129]
Casco Bay	USA	NAM	TNA	Lazzari [130]
Casco Bay	USA	NAM	TNA	Lazzari [130]
Weskeag River	USA	NAM	TNA	Lazzari [130]
Weskeag River	USA	NAM	TNA	Lazzari [130]
Barataria Bay - Caminada Bay	USA	NAM	TNA	Granados-Dieseldorff and Baltz [131]
Aransas Bay - San Antonio Bay	USA	NAM	TNA	Rozas and Minello [132]
Chesapeake Bay	USA	NAM	TNA	Weinstein and Brooks [133]
Puget Sound	USA	NAM	TNP	Toft, Cordell, Simenstad and Stamatiou [134]
Alamitos Bay	USA	NAM	TNP	Valle, O´Brien and Wiese [135]
Great Bay (NJ)	USA	NAM	TNA	Szedlmayer and Able [136]
Tijuana	USA	NAM	TNP	Nordby and Zedler [137]
Los Penasquitos Lagoon	USA	NAM	TNP	Nordby and Zedler [137]
Wells	USA	NAM	TNA	Ayvazian, Deegan and Finn [138]

Waquoit Bay	USA	NAM	TNA	Ayvazian, Deegan and Finn [138]
Newport Bay (NAM)	USA	NAM	TNP	Allen [139]
Morro Bay	USA	NAM	TNP	Horn [140]
Alamitos Bay	USA	NAM	TNP	Allen and Horn [141]
Yaquina Bay	USA	NAM	TNP	Bayer [142]
Tillamook Bay	USA	NAM	TNP	Bottom and Forsberg [143]
Tillamook Bay	USA	NAM	TNP	Bottom and Forsberg [143]
Umpqua River	USA	NAM	TNP	Bottom, Miller and Jones [144]
Tillamook Bay	USA	NAM	TNP	Cummings and Berry [145]
Tillamook Bay	USA	NAM	TNP	Ellis [146]
Chesapeake Bay	USA	NAM	TNA	Ruiz, Hines and Posey [147]
Chesapeake Bay	USA	NAM	TNA	Ruiz, Hines and Posey [147]
Puget Sound	USA	NAM	TNP	Fresh [148]
San Diego Bay	USA	NAM	TNP	Allen, Findlay and Phalen [149]
Tijuana	USA	NAM	TNP	Williams, West and Zedler [150]
Los Penasquitos Lagoon	USA	NAM	TNP	Williams, West and Zedler [150]
San Diego Bay	USA	NAM	TNP	Williams and Zedler [151]
Old Fort Bayou - Biloxi Bay	USA	NAM	TNA	Peterson and Ross [152]
St Catherine Sound - Sapelo Sound	USA	NAM	TNA	Dahlberg and Odum [153]
Chesapeake Bay	USA	NAM	TNA	Merriner, Kriete and Grant [154]
Great Bay (NJ)	USA	NAM	TNA	Rountree [155]
North Edisto	USA	NAM	TNA	Crabtree and Dean [156]
North Edisto	USA	NAM	TNA	Crabtree and Dean [156]
Humboldt Bay	USA	NAM	TNP	Garwood, Mulligan and Bjorkstedt [157]
St Catherine Sound - Sapelo Sound	USA	NAM	TNA	Dahlberg [158]
Apalachicola Bay	USA	NAM	TNA	Gorecki and Davis [159]
Apalachicola Bay	USA	NAM	TNA	Livingston [160]
Pamlico Sound	USA	NAM	TNA	Mabe [161]
Pamlico Sound	USA	NAM	TNA	Ross and Epperly [162]
Pamlico Sound	USA	NAM	TNA	Kirby-Smith, Lebo and Herrmann [163]
Santee Rivers	USA	NAM	TNA	Wenner, Shealy Jr. and Sandifer [164]
Biscayne Bay	USA	NAM	TAt	Serafy, Lindeman, Hopkins and Ault [165]
Lower Laguna Madre	USA	NAM	TNA	Hook [166]
Narragansett Bay	USA	NAM	TNA	Oviatt and Nixon [167]
Carpinteria	USA	NAM	TNP	Brooks [168]
Pensacola Bay	USA	NAM	TNA	Lewis, Goodman, Chancy and Jordan [169]
St Mary	USA	NAM	TNA	Solomon, Brodie and Ehlinger [170]
Pensacola Bay	USA	NAM	TNA	Cooley [171]
St Andrew Bay	USA	NAM	TNA	Ogren and Brusher [172]
St Paul Inlet and Bay	Canada	NAM	TNA	Melanson and Campbell [173]
Bonne Bay	Canada	NAM	TNA	Currie, Wroblewski, Methven and Hooper [174]
Bonne Bay	Canada	NAM	TNA	Currie, Wroblewski, Methven and Hooper [174]
Bonne Bay	Canada	NAM	TNA	Currie, Wroblewski, Methven and Hooper [174]
North Edisto	USA	NAM	TNA	Shealy Jr., Miglarese and Joseph [175]
Pamlico Sound	USA	NAM	TNA	Rulifson [176]
Pamlico Sound	USA	NAM	TNA	Rulifson [176]
Pamlico Sound	USA	NAM	TNA	Purvis [177]
Westport	USA	NAM	TNA	Fiske, Curley and Lawton [178]

Narragansett Bay	USA	NAM	TNA	Meng and Powell [179]
Aransas Bay - San Antonio Bay	USA	NAM	TNA	Moore [180]
Sheepscot River and Back River	USA	NAM	TNA	Targett and McCleave [181]
Penobscot Bay	USA	NAM	TNA	Lazzari and Tupper [182]
Cook Inlet	USA	NAM	TNP	Robards, Piatt, Kettle and Abookire [183]
Long Island Sound	USA	NAM	TNA	Hillman, Davis and Wennemer [184]
Estero de Punta Banda	Mexico	NAM	TNP	Rosales-Casián [185]
St Marks	USA	NAM	TNA	Subrahmanyam and Coultas [186]
Savannah	USA	NAM	TNA	Jennings and Weyers [187]
San Francisco Bay	USA	NAM	TNP	Kimmerer, Gross and MacWilliams [188]
San Francisco Bay	USA	NAM	TNP	Kimmerer, Gross and MacWilliams [188]
Patos lagoon	Brasil	SAM	TSAm	Garcia and Vieira [189]
Mampituba estuary	Brasil	SAM	TSAm	Ramos and Vieira [190]
Peixe lagoon	Brasil	SAM	TSAm	Ramos and Vieira [190]
Patos lagoon	Brasil	SAM	TSAm	Ramos and Vieira [190]
Chui estuary	Brasil	SAM	TSAm	Ramos and Vieira [190]
La plata river estuary	Argentina	SAM	TSAm	Jaureguizar, Menni, Guerrero and Lasta [191]
Caete estuary	Brasil	SAM	TAt	Barletta, Barletta-Bergan, Saint-Paul and Hubold [192]
Peixe lagoon	Brasil	SAM	TSAm	Loebmann and Vieira [193]
Peixe lagoon	Brasil	SAM	TSAm	Loebmann and Vieira [193]
Peixe lagoon	Brasil	SAM	TSAm	Loebmann and Vieira [193]
Peixe lagoon	Brasil	SAM	TSAm	Loebmann and Vieira [193]
Sai Guacu river estuary	Brasil	SAM	TSAm	Vendel and Chaves [194]
Champoton estuary	Mexico	NAM	TAt	López-López, Elías Sedeño-Díaz, Romero and Trujillo-Jiménez [195]
Formoso river estuary	Brasil	SAM	TAt	de Paiva, Lima, de Souza and de Araújo [196]
Curuca river estuary	Brasil	SAM	TAt	Vilar, et al. [197]
Paranagua bay	Brasil	SAM	TSAm	Vilar, et al. [197]
Piraque-acu estuary	Brasil	SAM	TAt	Vilar, et al. [197]
Paranagua bay	Brasil	SAM	TSAm	Vilar, et al. [197]
Chacahua lagoon	Mexico	NAM	TEP	Mendoza, Castillo-Rivera, Zárate-Hernández and Ortiz-Burgos [198]
Paranagua bay	Brasil	SAM	TSAm	Contente, Stefanoni and Spach [199]
Guaratuba bay	Brasil	SAM	TSAm	Vendel, Bouchereau and Chaves [200]
Magdalena river delta	Colombia	SAM	TAt	Rueda and Defeo [202]
Sepetiba bay	Brasil	SAM	TSAm	Loebmann and Vieira [203]
Sepetiba bay	Brasil	SAM	TSAm	Araújo, da Cruz-Filho, de Azevêdo and Santos [204]
Pueblo Viejo estuary	Mexico	NAM	TAt	Castillo-Rivera, Zavala-Hurtado and Zárate [205]
Pueblo Viejo estuary	Mexico	NAM	TAt	Castillo-Rivera, Ortiz-Burgos and Zárate-Hernández [206]
Mar Chiquita lagoon	Argentina	SAM	TSAm	González Castro, et al. [207]
Peixe Lagoon	Brasil	SAM	TSAm	Loebmann, et al. [208]
Mar Chiquita lagoon	Argentina	SAM	TSAm	Loebmann, et al. [208]
Terminos lagoon	Mexico	NAM	TAt	Ramos-Miranda, et al. [209]
Mambucaba estuary	Brasil	SAM	TSAm	Neves, Teixeira and Araújo [210]
Magdalena river delta	Colombia	SAM	TAt	Ramírez and Rueda [211]
Magdalena river delta	Colombia	SAM	TAt	Ramírez and Rueda [211]
Magdalena river delta	Colombia	SAM	TAt	Ramírez and Rueda [211]
Babitonga bay	Brasil	SAM	TSAm	Vilar, Spach and Souza-Conceição [213], Vilar, Spach and Joyeux [214]
Babitonga bay	Brasil	SAM	TSAm	Vilar, Spach and Santos [215]
Sai Guacu river estuary	Brasil	SAM	TSAm	Spach, et al. [216]

Paranagua bay	Brasil	SAM	TSAm	Schwarz Jr., et al. [217]
Paranagua bay	Brasil	SAM	TSAm	Santos, Schwarz Jr., de Oliveira Neto and Spach [218]
Joanes River Estuary	Brasil	SAM	TAt	Reis-Filho, Nunes, de Menezes and de Souza [219]
Todos os Santos bay	Brasil	SAM	TAt	de Oliveira-Silva, Peso-Aguiar and Lopes [220]
Contas river estuary	Brasil	SAM	TAt	Lima [221]
Paranagua bay	Brasil	SAM	TSAm	Falcão, et al. [222]
Paranagua bay	Brasil	SAM	TSAm	Falcão, et al. [222]
Terminos lagoon	Mexico	NAM	TAt	Arceo-Carranza, Vega-Cendejas, Montero-Muñoz and de Santillana [224]
Coto-colorado estuary	Costa Rica	NAM	TEP	Feutry, Hartmann, Casabonnet and Umaña [225]
Jiquilisco bay	El Salvador	NAM	TEP	Phillips [226]
Curuca river estuary	Brasil	SAM	TAt	Hercos [227]
Huizache-Caimanero lagoon system	Mexico	NAM	TNP	Amezcua-Linares [228]
Huizache-Caimanero lagoon system	Mexico	NAM	TNP	Warburton [229]
Apozahualco lagoon	Mexico	NAM	TEP	Yáñez-Arancibia [230]
Chautengo lagoon	Mexico	NAM	TEP	Yáñez-Arancibia [230]
Tecomate lagoon	Mexico	NAM	TEP	Yáñez-Arancibia [230]
Tres Palos lagoon	Mexico	NAM	TEP	Yáñez-Arancibia [230]
Coyuca lagoon	Mexico	NAM	TEP	Yáñez-Arancibia [230]
Mitla lagoon	Mexico	NAM	TEP	Yáñez-Arancibia [230]
Nuxco lagoon	Mexico	NAM	TEP	Yáñez-Arancibia [230]
Potosi lagoon	Mexico	NAM	TEP	Yáñez-Arancibia [230]
Guanabara bay	Brasil	SAM	TSAm	Rodrigues, Lavrado, Falcão and da Silva [231]
Sul bay- Florinopolis	Brasil	SAM	TSAm	Cartagena, Hostim-Silva and Spach [232]
Sepetiba bay	Brasil	SAM	TSAm	Pessanha and Araújo [233]
Paranagua bay	Brasil	SAM	TSAm	Hackradt, et al. [234]
Magdalena river delta	Colombia	SAM	TAt	Álvarez-León [235]
Gulf of Nicoya	Mexico	NAM	TEP	Bartels, Price, López and Bussing [236]
El verde coastal lagoon	Mexico	NAM	TNP	Gonzalez [237]
Tamiahua lagoon	Mexico	NAM	TAt	Diaz-Ruiz, Pérez-Hernández and Aguirre-León [238]
Chantuto-Panzacola estuarine system	Mexico	NAM	TEP	Díaz-Ruiz, Cano-Quiroga, Aguirre-León and Ortega-Bernal [239]
Tamiahua lagoon	Mexico	NAM	TAt	Franco-López and Chavez-López [240]
Gulf of Nicoya	Mexico	NAM	TEP	León [241]
Mazatlan	Mexico	NAM	TNP	León [242]
Mar Muerto lagoon	Mexico	NAM	TEP	Tapia-García, Núñez, de Guevara, Montes and Abad [243]
Mar Muerto lagoon	Mexico	NAM	TEP	Tapia-García, Núñez, de Guevara, Montes and Abad [243]
Mar Muerto lagoon	Mexico	NAM	TEP	Núñez [244]
Magdalena river delta	Colombia	SAM	TAt	Vera and Muñoz [245]

Table S2. Summary of variables included in the database.

Variable	Description	Type	Unit (if numeric) or levels (if categorical)	Spatial resolution	Source
Marine Biogeographic Realm	marine biogeographic realm sensu Spalding, et al. [8]	Categorical	TSAf - Temperate Southern Africa, TNA - Temperate Northern Atlantic, TNP - Temperate Northern Pacific, TSAm - Temperate South America, TAu - Temperate Australasia, TAt - Tropical Atlantic, TEP - Tropical Eastern Pacific, WIP - Western Indo-Pacific, CIP - Central Indo-Pacific	estuary scale	data from Spalding, et al. [8] freely available at www.marineregions.org [247]
Continent	continent	Categorical	NAM - North America, SAM - South America, EUR - Europe, AFR - Africa, ASI - Asia, OCE - Oceania. Total number of estuaries in the database is respectively: 74, 21, 108, 116, 8, 51. Total number of samples in the database is respectively: 116, 44, 140, 152, 10, 68.	estuary scale	estimated using shapefiles in ArcGIS [7]
Sea Surface Temperature	sea surface temperature at the estuary mouth (mean values estimated based on monthly data from 2002 to 2009).	Numerical	ºC	5 arcmin	estimated using shapefiles in ArcGIS [7]; data freely available at www.oracle.ugent.be [9]
Terrestial net primary productivity	net amount of carbon the plants take up in an average square meter of the grid cell during an average year, based on 135 year model simulation (1860-1994).	Numerical	$gC.m^{-2}.day^{-1}$	1 degree	estimated using shapefiles in ArcGIS [7]; data from Foley, et al. [10] and Kucharik, et al. [11] freely available at https://nelson.wisc.edu/sage/
Chlorophyll a concentration	mean chlorophyll a concentration at the estuary mouth (mean values estimated based on monthly data from 2002 to 2009).	Numerical	$mg.m^3$	5 arcmin	estimated using shapefiles in ArcGIS [7]; data freely available at www.oracle.ugent.be [9]

Table S2. (cont)

Variable	Description	Type	Unit (if numeric) or levels (if categorical)	Spatial resolution	Source
Continental shelf width	minimum distance to continental shelf limit (150m bathymetry) measured from the mouth of each estuary.	Numerical	m	estuary scale	estimated using shapefiles in ArcGIS [7]
Tidal range	macrotidal (>4m); mesotidal (2-4m); microtidal (0-2m).	Ordinal	(1) microtidal, (2) mesotidal, (3) macrotidal	estuary scale	the original publication on the fish assemblage for an estuary, and other publications
Estuary type	Connectivity of the estuary with the adjacent marine ecosystem: open or temporarily open.	Ordinal	(1) temporarily open, (2) open	estuary scale	the original publication on the fish assemblage for an estuary, and other publications
Estuary mouth width	Total width of the mouth/s of an estuary.	Numerical	m	estuary scale	estimated using shapefiles in ArcGIS [7]
Estuary area	total area of the estuary.	Numerical	km^2	estuary scale	the original publication on the fish assemblage for an estuary, and other publications
Drainage basin area	total area of the drainage basin.	Numerical	km^2	estuary scale	the original publication on the fish assemblage for an estuary, and other publications
Estuary salinity type	regular (typically <40), regular to hyperhaline (occasionally with areas >40), hyperhaline (frequently with areas >40).	Ordinal	(1) regular, (2) regular to hyperhaline, (3) hyperhaline	estuary scale	the original publication on the fish assemblage for an estuary, and other publications

Table S3. Pairwise Pearson correlation between traits of fish assemblages in a set of estuaries distributed worldwide: body size, vulnerability and resilience (relative taxa richness of trait categories). ns - non significant at p<0.05. Maximum body size reflects many aspects such as position in the food web, species abundance, metabolic rates, dispersal ability and home range; small (<15cm), medium (15-50cm), large (50-100), very large (>100cm). (total number of samples is 530, for a total of 378 estuaries).

		Body size				Fish vulnerability					Fish resilience			
		S	M	L	VL	L	L-M	M-H	H-VH	VH	H	M	L	VL
Body size	Small (S)													
	Medium (M)	-0.4												
	Large (L)	-0.6	-0.2											
	Very large (VL)	-0.4	-0.2	0.1										
Vulnerability	Low (L)	0.7	ns	-0.6	-0.5									
	Low-Moderate (L-M)	-0.4	0.2	0.2	0.1	-0.5								
	Moderate-High (M-H)	-0.4	-0.1	0.5	0.3	-0.7	ns							
	High-Very high (H-VH)	-0.4	-0.1	0.6	0.3	-0.5	0.2	0.2						
	Very high (VH)	-0.2	-0.1	ns	0.6	-0.4	ns	0.2	ns					
Resilience	High (H)	0.7	ns	-0.6	-0.5	0.9	-0.4	-0.6	-0.5	-0.4				
	Medium (M)	-0.6	ns	0.6	0.4	-0.7	0.6	0.5	0.6	0.1	-0.8			
	Low (L)	-0.2	ns	0.2	0.3	-0.4	0.1	0.4	0.1	0.4	-0.4	ns		
	Very Low (VL)	-0.4	ns	0.3	0.3	-0.4	ns	0.2	0.3	0.5	-0.4	0.2	ns	

3. Supplementary figures

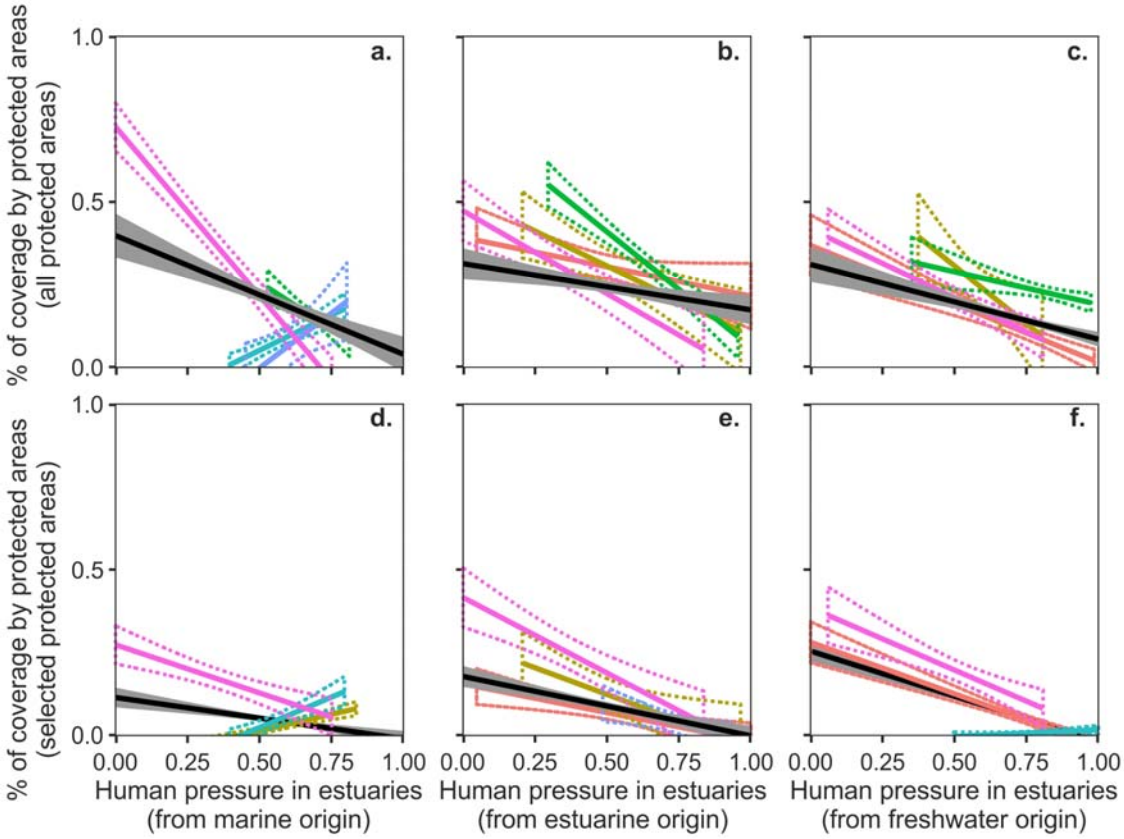

Figure S1. Linear relationships (and 95% confidence intervals) between human pressure and percentage of coverage by protected areas in and around estuaries distributed worldwide. Human pressures and protection were assessed for: marine ecosystem (first column, directly in estuaries (second column), and for freshwater ecosystem (third column). Coverage by protected areas was determined in two ways: considering all protected areas (top row) and selected protected areas with IUCN management categories I-IV (bottom row). Linear relationships are presented for all estuaries (●) and per continent: North America (●), South America (●), Europe (●), Africa (●), Asia (●), Oceania (●). Only significant linear relationships are represented ($P<0.05$). All variables were log-transformed, and pressure variables were also normalized.

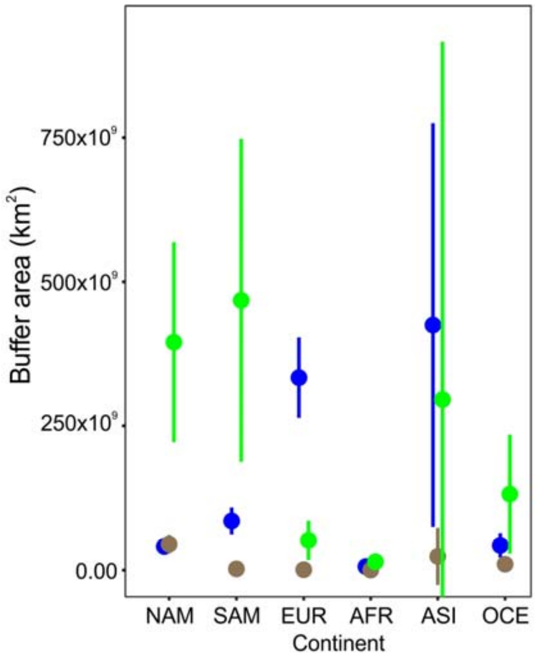

Figure S2. Mean area (and 95% confidence interval) of influence circles (in km^2) defined in the present study for the estimation of the intensity of human activity and pressure as well as the percentage of coverage by protected areas, affecting estuaries distributed worldwide. Values are presented per ecosystem (● marine, ● estuarine, ● freshwater) and per continent (NAM - North America, SAM - South America, EUR - Europe, AFR - Africa, ASI - Asia, OCE - Oceania). (total number of samples is 530, for a total of 378 estuaries).

References

1 Potter, I. C., Chuwen, B. M., Hoeksema, S. D. & Elliott, M. The concept of an estuary: A definition that incorporates systems which can become closed to the ocean and hypersaline. *Estuar Coast Shelf Sci* **87**, 497-500, (2010).

2 Pritchard, R. W. in *Estuaries* (ed G.H. Lauff) 3-5 (American Association for the Advancement of Science, Washington D.C., 1967).

3 Day, J. H. What is an estuary? *South African Journal of Science* **76**, 198, (1980).

4 Elliott, M. & McLusky, D. S. The Need for Definitions in Understanding Estuaries. *Estuar Coast Shelf Sci* **55**, 815-827, (2002).

5 Whitfield, A. K., Elliott, M., Basset, A., Blaber, S. J. M. & West, R. J. Paradigms in estuarine ecology - A review of the Remane diagram with a suggested revised model for estuaries. *Estuar Coast Shelf Sci* **97**, 78-90, (2012).

6 Froese, F. & Pauly, D. FishBase, www.fishbase.org, (2014) (Date of access 01/01/2014)

7 ESRI. ArcGIS for Desktop, http://desktop.arcgis.com, v. 10.4 (ESRI, 2016).

8 Spalding, M. D. *et al.* Marine ecoregions of the world: A bioregionalization of coastal and shelf areas. *Bioscience* **57**, 573-583, (2007).

9 Tyberghein, L. *et al.* Bio-ORACLE: a global environmental dataset for marine species distribution modelling. *Glob Ecol Biogeogr* **21**, 272-281, (2012).

10 Foley, J. A. *et al.* An integrated biosphere model of land surface processes, terrestrial carbon balance, and vegetation dynamics. *Global Biogeochemical Cycles* **10**, 603-628, (1996).

11 Kucharik, C. J. *et al.* Testing the performance of a dynamic global ecosystem model: Water balance, carbon balance, and vegetation structure. *Global Biogeochemical Cycles* **14**, 795-825, (2000).

12 Wasserman, R. J. & Strydom, N. A. The importance of estuary head waters as nursery areas for young estuary- and marine-spawned fishes in temperate South Africa. *Estuar Coast Shelf S* **94**, 56-67, (2011).

13 Bailey, S. E. & James, N. C. Fish sampling in the marine-dominated Kariega Estuary, South Africa, using a demersal otter trawl: day/night effects. *Afr J Aquat Sci* **38**, 115-120, (2013).

14 Whitfield, A. K. & Paterson, A. W. Distribution patterns of fishes in a freshwater deprived Eastern Cape estuary, with particular emphasis on the geographical headwater region. *Water SA* **29**, 61-68, (2003).

15 Lukey, J. R., Booth, A. J. & Froneman, P. W. Fish population size and movement patterns in a small intermittently open South African estuary. *Estuar Coast Shelf S* **67**, 10-20, (2006).

16 James, N. C., Whitfield, A. K. & Cowley, P. D. Long-term stability of the fish assemblages in a warm-temperate South African estuary. *Estuar Coast Shelf S* **76**, 723-738, (2008).

17 Vivier, L., Cyrus, D. P., Owen, R. K. & Jerling, H. L. Fish assemblages in the Mfolozi–Msunduzi estuarine system, KwaZulu-Natal, South Africa, when not linked to the St Lucia mouth. *Afr J Aquat Sci* **35**, 141-154, (2010).

18 Withfield, A. K. & Harrison, T. D. River flow and fish abundance in a South African estuary. *J Fish Biol* **62**, 1467-1472, (2003).

19 Simier, M., Blanc, L., Aliaume, C., Diouf, P. S. & Albaret, J. J. Spatial and temporal structure of fish assemblages in an “inverse estuary”, the Sine Saloum system (Senegal). *Estuar Coast Shelf S* **59**, 69-86, (2004).

20 Faye, D. *et al.* Structure and seasonal variability of fish food webs in an estuarine tropical marine protected area (Senegal): Evidence from stable isotope analysis. *Estuar Coast Shelf S* **92**, 607-617, (2011).

21 Huxham, M., Kimani, E. & Augley, J. Mangrove fish: a comparison of community structure between forested and cleared habitats. *Estuar Coast Shelf S* **60**, 637-647, (2004).

22 Crona, B. I. & Rönnbäck, P. Community structure and temporal variability of juvenile fish assemblages in natural and replanted mangroves, *Sonneratia alba* Sm., of Gazi Bay, Kenya. *Estuar Coast Shelf S* **74**, 44-52, (2007).

23 de Troch, M., Mees, J., Papadopoulos, I. & Wakwabi, E. O. Fish communities in a tropical bay (Gazi Bay, Kenya): seagrass beds vs. unvegetated areas. *Neth J Biol* **46**, 236-252, (1996).

24 Huxham, M., Kimani, E. & Augley, J. The Fish Community of an East African Mangrove: Effects of Turbidity and Distance from the Sea. *WIO J. Mar. Sci.* **7**, 57-67, (2008).
25 Ecoutin, J.-M., Richard, E., Simier, M. & Albaret, J.-J. Spatial versus temporal patterns in fish assemblages of a tropical estuarine coastal lake: The Ebrié Lagoon (Ivory Coast). *Estuar Coast Shelf S* **64**, 623-635, (2005).
26 Harrison, T. D. *Ecology of the ichthyofauna in three temporarily open/closed estuaries on the Natal Coast* MSc Thesis thesis, Rhodes University, (1993).
27 Mohamed, A.-R. M., Resen, A. K. & Taher, M. M. Longitudinal patterns of fish community structure in the Shatt Al-Arab River, Iraq. *Basrah Journal of Science* **30**, 65-86, (2012).
28 Grant, W. L. *The community structure and feeding ecology of the icthyofauna in the Mngazana and Mngazi estuaries, Port St. Johns, South Africa* MSc Thesis thesis, Faculty of Science, Nelson Mandela Metropolitan University, (2007).
29 Beckley, L. E. The Ichthyofauna of the Sundays Estuary, South Africa, with Particular Reference to the Juvenile Marine Component. *Estuaries* **7**, 248-258, (1984).
30 Harrison, T. D. *Biogeography and community structure of fishes in South African estuaries* PhD Thesis thesis, Rhodes University, (2003).
31 Vorwerk, P. D. *Ichthyofaunal community structures in different types of Eastern Cape Estuaries* MSc Thesis thesis, Rhodes University, (2000).
32 Quan, W., Shi, L. & Chen, Y. Comparison of Nekton Use for Cordgrass *Spartina alterniflora* and Bulrush *Scirpus mariqueter* Marshes in the Yangtze River Estuary, China. *Estuaries Coasts* **34**, 405-416, (2011).
33 Tongnunui, P. *et al.* Fish fauna of the Sikao Creek mangrove estuary, Trang, Thailand. *Fisheries Sci* **68**, 10-17, (2002).
34 Shinnaka, T. *et al.* Effects of mangrove deforestation on fish assemblage at Pak Phanang Bay, southern Thailand. *Fisheries Sci* **73**, 862-870, (2007).
35 Tachihara, K. *et al.* Ichthyofauna in Mangrove Estuaries of the Okinawa, Miyako, Ishigaki and Iriomote Islands during August from 2000 to 2002. *Bull. Soc. Sea Water Sci. Jpn.* **57**, 481-490, (2003).
36 Rashed-Un-Nabi, M., Al-Mamun, M. A., Ullah, M. H. & Mustafa, M. G. Temporal and spatial distribution of fish and shrimp assemblage in the Bakkhali river estuary of Bangladesh in relation to some water quality parameters. *Mar Biol Res* **7**, 436-452, (2011).
37 Hajisamae, S., Yeesin, P. & Chaimongkol, S. Habitat utilization by fishes in a shallow, semi-enclosed estuarine bay in southern Gulf of Thailand. *Estuar Coast Shelf S* **68**, 647-655, (2006).
38 Kanou, K., Sano, M. & Kohno, H. Larval and juvenile fishes occurring with flood tides on an intertidal mudflat in the Tama River estuary, central Japan. *Ichthyol Res* **52**, 158-164, (2005).
39 Pinto, L. & Punchihewa, N. N. Utilisation of mangroves and seagrasses by fishes in the Negombo Estuary, Sri Lanka. *Mar Biol* **126**, 333-345, (1996).
40 Wang, M., Huang, Z., Shi, F. & Wang, W. Are vegetated areas of mangroves attractive to juvenile and small fish? The case of Dongzhaigang Bay, Hainan Island, China. *Estuar Coast Shelf S* **85**, 208-216, (2009).
41 Kanou, K., Koike, T. & Kohno, H. Ichthyofauna of tidelands in the inner Tokyo Bay, and its diversity. *JPN J Ichthy* **47**, 115-129, (2000).
42 Hermosilla, J. J., Tamura, Y., Moteki, M. & Kohno, H. Distribution and community structure of fish in Obitsu-gawa River Estuary of inner Tokyo Bay, central Japan. *AACL Bioflux* **5**, 197-222, (2012).
43 Griffiths, S. P. Diel variation in the seagrass ichthyofunas of three intermittently open estuaries in south-eastern Australia: implications for improving fish diversity assessments. *Fish Manag Ecol* **8**, 123-140, (2001b).
44 Griffiths, S. P. Factors Influencing Fish Composition in an Australian Intermittently Open Estuary. Is Stability Salinity-Dependent? *Estuar Coast Shelf S* **52**, 739-751, (2001a).
45 Hoeksema, S. D. & Potter, I. C. Diel, seasonal, regional and annual variations in the characteristics of the ichthyofauna of the upper reaches of a large Australian microtidal estuary. *Estuar Coast Shelf S* **67**, 503-520, (2006).
46 Kanandjembo, A. N., Potter, I. C. & Platell, M. E. Abrupt shifts in the fish community of the hydrologically variable upper estuary of the Swan River. *Hydrol Process* **15**, 2503-2517, (2001).

47 Loneragan, N. R., Potter, I. C. & Lenanton, R. C. J. Influence of site, season and year on contributions made by marine, estuarine, diadromous and freshwater species to the fish fauna of a temperate Australian estuary. *Mar Biol* **103**, 461-479, (1989).
48 Valesini, F. J., Potter, I. C., Platell, M. E. & Hyndes, G. A. Ichthyofaunas of a temperate estuary and adjacent marine embayment. Implications regarding choice of nursery area and influence of environmental changes. *Mar Biol* **128**, 317-328, (1997).
49 Akin, S., Buhan, E., Winemiller, K. O. & Yilmaz, H. Fish assemblage structure of Koycegiz Lagoon–Estuary, Turkey: Spatial and temporal distribution patterns in relation to environmental variation. *Estuar Coast Shelf S* **64**, 671-684, (2005).
50 Vance, D. J. *et al.* How far do prawns and fish move into mangroves? Distribution of juvenile banana prawns *Penaeus merguiensis* and fish in a tropical mangrove forest in northern Australia. *Mar Ecol Prog Ser* **131**, 115-124, (1996).
51 Bloomfield, A. L. & Gillanders, B. M. Fish and Invertebrate Assemblages in Seagrass, Mangrove, Saltmarsh, and Nonvegetated Habitats. *Estuaries* **28**, 63-77, (2005).
52 Jackson, G. & Jones, G. K. Spatial and temporal variation in nearshore fish and macroinvertebrate assemblages from a temperate Australian estuary over a decade. *Mar Ecol Prog Ser* **182**, 253-268, (1999).
53 Jones, G. K., Baker, J. L., Edyvane, K. & Wright, G. J. Nearshore Fish Community of the Port River-Barker Inlet Estuary, South Australia. I. Effect of Thermal Effluent on the Fish Community Structure, and Distribution and Growth of Economically Important Fish Species. *Mar Fresh Res* **47**, 785-799, (1996).
54 Gray, C. A., McElligott, D. J. & Chick, R. C. Intra- and Inter-estuary Differences in Assemblages of Fishes Associated with Shallow Seagrass and Bare Sand. *Mar Fresh Res* **47**, 723-735, (1996).
55 Rotherham, D., Broadhurst, M. K., Gray, C. A. & Johnson, D. D. Developing a beam trawl for sampling estuarine fish and crustaceans: assessment of a codend cover and effects of different sizes of mesh in the body and codend. *ICES J Mar Sci* **65**, 687-696, (2008).
56 West, R. J. Comparison of fish and shrimp trawls for sampling deep-water estuarine fish in a large coastal river in eastern Australia. *Fish Res* **54**, 409-417, (2002).
57 Rotherham, D., Gray, C. A., Johnson, D. D. & Lokys, P. Effects of diel period and tow duration on estuarine fauna sampled with a beam trawl over bare sediment: Consequences for designing more reliable and efficient surveys. *Estuar Coast Shelf S* **78**, 179-189, (2008).
58 Griffiths, S. P. & West, R. J. Preliminary assessment of shallow water fish in three small intermittently open estuaries in south-eastern Australia. *Fish Manag Ecol* **6**, 311-321, (1999).
59 Hoeksema, S. D., Chuwen, B. M. & Potter, I. C. Comparisons between the characteristics of ichthyofaunas in nearshore waters of five estuaries with varying degrees of connectivity with the ocean. *Estuar Coast Shelf S* **85**, 22-35, (2009).
60 Humphries, P., Potter, I. C. & Loneragan, N. R. The Fish Community in the Shallows of a Temperate Australian Estuary: Relationships with the Aquatic Macrophyte *Ruppia megacarpa* and Environmental Variables. *Estuar Coast Shelf S* **34**, 325-346, (1992).
61 Potter, I. C., Hyndes, G. A. & Baronie, F. M. The fish fauna of a seasonally closed Australian estuary. Is the prevalence of estuarine-spawning species high? *Mar Biol* **116**, 19-30, (1993).
62 Potter, I. C. & Hyndes, G. A. Composition of the fish fauna of a permanently open estuary on the southern coast of Australia, and comparisons with a nearby seasonally closed estuary. *Mar Biol* **121**, 199-209, (1994).
63 Potter, I. C., Loneragan, N. R., Lenanton, R. C. J., Chrystal, P. J. & Grant, C. J. Abundance, distribution and age structure of fish populations in a Western Australian estuary. *J. Zool.* **200**, 21-50, (1983).
64 Young, G. C. & Potter, I. C. Induction of annual cyclical changes in the ichthyofauna of a large microtidal estuary following an artificial and permanent increase in tidal flow. *J Fish Biol* **63**, 1306-1330, (2003).
65 Morrison, M. A., Francis, M. P., Hartill, B. W. & Parkinson, D. M. Diurnal and Tidal Variation in the Abundance of the Fish Fauna of a Temperate Tidal Mudflat. *Estuar Coast Shelf S* **54**, 793-807, (2002).
66 Morton, R. M. Community structure, density and standing crop of fishes in a subtropical Australian mangrove area. *Mar Biol* **105**, 385-394, (1990).

67 Quinn, N. J. Analysis of temporal changes in fish assemblages in Serpentine Creek, Queensland. *Environ Biol Fish* **5**, 117-133, (1980).
68 Rotherham, D. & West, R. J. Do different seagrass species support distinct fish communities in south-eastern Australia. *Fish Manag Ecol* **9**, 235-248, (2002).
69 Sheaves, M. & Johnston, R. Ecological drivers of spatial variability among fish fauna of 21 tropical Australian estuaries. *Mar Ecol Prog Ser* **385**, 245-260, (2009).
70 Young, G. C., Potter, I. C., Hyndes, G. A. & de Lestang, S. The Ichthyofauna of an Intermittently Open Estuary: Implications of Bar Breaching and Low Salinities on Faunal Composition. *Estuar Coast Shelf S* **45**, 53-68, (1997).
71 Potter, I. C., Tiivel, D., Valesini, F. J. & Hyndes, G. A. Comparisons between the ichthyofaunas of a temperate lagoonal-like estuary and the embayment into wich that estuary discharges. *Int. J. Salt Lake Res.* **5**, 337-358, (1997).
72 Blaber, S. J. M. Fish of the Trinity Inlet System of North Queensland with Notes on the Ecology of Fish Faunas of Tropical Indo-Pacific Estuaries. *Aust. J. Mar. Freshw. Res.* **31**, 137-146, (1980).
73 Pasquaud, S. *et al.* Impact of the sampling protocol in assessing ecological trends in an estuarine ecosystem: The empirical example of the Gironde estuary. *Ecol Indic* **15**, 18-29, (2012).
74 Cardoso, I. *et al.* Fish assemblages of small estuaries of the Portuguese coast: A functional approach. *Estuar Coast Shelf S* **93**, 40-46, (2011).
75 Costa-Dias, S., Freitas, V., Sousa, R. & Antunes, C. Factors influencing epibenthic assemblages in the Minho Estuary (NW Iberian Peninsula). *Mar Pollut Bull* **61**, 240-246, (2010).
76 França, S., Costa, M. J. & Cabral, H. N. Inter- and intra-estuarine fish assemblage variability patterns along the Portuguese coast. *Estuar Coast Shelf S* **91**, 262-271, (2011).
77 Matič-Skoko, S., Peharda, M., Pallaoro, A., Cukrov, M. & Baždarič, B. Infralittoral fish assemblages in the Zrmanja estuary, Adriatic Sea. *Acta Adriatica* **48**, 45-55, (2007).
78 Thiel, R., Cabral, H. & Costa, M. J. Composition, temporal changes and ecological guild classification of the ichthyofaunas of large European estuaries - a comparison between the Tagus (Portugal) and the Elbe (Germany). *J. Appl. Ichthyol.* **19**, 330-342, (2003).
79 Selleslagh, J. & Amara, R. Environmental factors structuring fish composition and assemblages in a small macrotidal estuary (eastern English Channel). *Estuar Coast Shelf S* **79**, 507-517, (2008).
80 Ribeiro, J. *et al.* Long-term changes in fish communities of the Ria Formosa coastal lagoon (southern Portugal) based on two studies made 20 years apart. *Estuar Coast Shelf S* **76**, 57-68, (2008).
81 Veiga, P., Vieira, L., Bexiga, C., Sá, R. & Erzini, K. Structure and temporal variations of fish assemblages of the Castro Marim salt marsh, southern Portugal. *Estuar Coast Shelf S* **70**, 27-38, (2006).
82 Gordo, L. S. & Cabral, H. N. The fish assemblage structure of a hydrologically altered coastal lagoon: the Óbidos lagoon (Portugal). *Hydrobiologia* **459**, 125-133, (2001).
83 Franco, A., Malavasi, S., Zucchetta, M., Franzoi, P. & Torricelli, P. Environmental influences on fish assemblage in the Venice Lagoon, Italy. *Chem Ecol* **22**, S105-S118, (2006).
84 Pombo, L., Rebelo, J. E. & Elliott, M. The structure, diversity and somatic production of the fish community in an estuarine coastal lagoon, Ria de Aveiro (Portugal). *Hydrobiologia* **587**, 253-268, (2007).
85 Marshall, S. & Elliott, M. The structure of the fish assemblage in the Humber estuary, U.K. *Publicaciones Especiales Instituto Español de Oceanografia* **21**, 231-242, (1996).
86 Pomfret, J. R., Turner, G. S. & Phillips, S. Beam trawl surveys as a monitoring tool in polluted estuaries in north-east England. *J Fish Biol* **33**, 71-77, (1988).
87 Martinho, F. *et al.* The influence of an extreme drought event in the fish community of a southern Europe temperate estuary. *Estuar Coast Shelf S* **75**, 537-546, (2007).
88 Araújo, F. G., Bailey, R. G. & Williams, W. P. Spatial and temporal variations in fish populations in the upper Thames estuary. *J Fish Biol* **55**, 836-853, (1999).
89 Costa, M. J. & Bruxelas, A. The structure of fish communities in the Tagus Estuary, Portugal, and its role as a nursery for commercial fish species. *Sci Mar* **53**, 561-566, (1989).

90 Elliott, M., O´Reilly, M. G. & Taylor, C. J. L. The Forth estuary: a nursery and overwintering area for North Sea fishes. *Hydrobiologia* **195**, 89-103, (1990).
91 Hamerlynck, O., Hostens, K., Arellano, R. V., Mees, J. & Van Damme, P. A. The mobile epibenthic fauna of soft bottoms in the Dutch Delta (South-West Netherlands): Spatial structure. *Neth J Aqua Ecol* **27**, 343-358, (1993).
92 Hostens, K. Spatial patterns and seasonality in the epibenthic communities of the Westerschelde (Southern Bight of the North Sea). *J Mar Biol Assoc UK* **80**, 27-36, (2000).
93 Koutrakis, E. T., Tsikliras, A. C. & Sinis, A. I. Temporal variability of the ichthyofauna in a Northern Aegean coastal lagoon (Greece). Influence of environmental factors. *Hydrobiologia* **543**, 245-257, (2005).
94 Mouillot, D., Dumay, O. & Tomasini, J. A. Limiting similarity, niche filtering and functional diversity in coastal lagoon fish communities. *Estuar Coast Shelf S* **71**, 443-456, (2007).
95 Ribeiro, J. *et al.* Seasonal, tidal and diurnal changes in fish assemblages in the Ria Formosa lagoon (Portugal). *Estuar Coast Shelf S* **67**, 461-474, (2006).
96 Selleslagh, J. *et al.* Fish composition and assemblage structure in three Eastern English Channel macrotidal estuaries: A comparison with other French estuaries. *Estuar Coast Shelf S* **81**, 149-159, (2009).
97 Uriarte, A. & Borja, A. Assessing fish quality status in transitional waters, within the European Water Framework Directive: Setting boundary classes and responding to anthropogenic pressures. *Estuar Coast Shelf S* **82**, 214-224, (2009).
98 Verdiell-Cubedo, D., Oliva-Paterna, F. J. & Torralva, M. Condition of *Gobius cobitis* (Pallas, 1811) juveniles in the Mar Menor coastal lagoon (SE Iberian Peninsula): Effects of inter- and intraspecific fish competition. *Sci Mar* **70**, 303-310, (2006).
99 Mouny, P., Dauvin, J. C., Bessincton, C., Elkaim, B. & Simon, S. Biological components from the Seine estuary: first results. *Hydrobiologia* **373/374**, 333-347, (1998).
100 Franzoi, P., Trisolini, R., Carrieri, A. & Rossi, R. Caratteristiche ecologiche del popolamento ittico ripario della Sacca di Scardovari (Delta del Po). *Nova Thalassia* **10**, 399-405, (1989).
101 Wilkońska, H. & Kapusta, A. Spatial and temporal variation in small fish occurrence in shallow habitats of the Vistula Lagoon (Southern Baltic Sea). *Acta Zool. Lit.* **17**, 203-212, (2007).
102 Nicolas, D. *et al.* Fish under influence: A macroecological analysis of relations between fish species richness and environmental gradients among European tidal estuaries. *Coast. Shelf Sci.* **86**, 137-147, (2010).
103 Mariani, S. Can Spatial Distribution of Ichthyofauna Describe Marine Influence on Coastal Lagoons? A Central Mediterranean Case Study. *Estuar Coast Shelf S* **52**, 261-267, (2001).
104 Hadderingh, R. H. & Jager, Z. Comparison of fish impingement by a thermal power station with fish populations in the Ems Estuary. *J Fish Biol* **61**, 105-124, (2002).
105 Nash, R. D. M. The Effects of Disturbance and Severe Seasonal Fluctuations in Environmental Conditions on North Temperate Shallow-water Fish Assemblages. *Estuar Coast Shelf S* **26**, 123-135, (1988).
106 De Ben, W. A., Clothier, W. D., Ditsworth, G. R. & Baumgartner, D. J. Spatio-Temporal Fluctuations in the Distribution and Abundance of Demersal Fish and Epibenthic Crustaceans in Yaquina Bay, Oregon. *Estuaries* **13**, 459-478, (1990).
107 Nybakken, J., Cailliet, G. & Broenkow, W. Ecologic and hydrographic studies of Elkhorn Slough Moss Landing harbor and nearshore coastal waters, July 1974 to June 1976. 480pp. (Moss Landing Marine Laboratories, Moss Landing, California, 1977).
108 Duffy, K. C. & Baltz, D. M. Comparison of fish assemblages associated with native and exotic submerged macrophytes in the Lake Pontchartrain estuary, USA. *J Exp Mar Biol Ecol* **223**, 199-221, (1998).
109 Desmond, J. S., Zedler, J. B. & Williams, G. D. Fish use of tidal creek habitats in two southern California salt marshes. *Ecological Engineering* **14**, 233-252, (2000).
110 Gelwick, F. P., Akin, S., Arrington, D. A. & Winemiller, K. O. Fish Assemblage Structure in Relation to Environmental Variation in a Texas Gulf Coastal Wetland. *Estuaries* **24**, 285-296, (2001).
111 Hagan, S. M. & Able, K. W. Seasonal changes of the pelagic fish assemblage in a temperate estuary. *Estuar Coast Shelf S* **56**, 15-29, (2003).

112 Jung, S. & Houde, E. D. Spatial and temporal variabilities of pelagic fish community structure and distribution in Chesapeake Bay, USA. *Estuar Coast Shelf S* **58**, 335-351, (2003).
113 Lazzari, M. A., Sherman, S. & Kanwit, J. K. Nursery use of shallow habitats by epibenthic fishes in Maine nearshore waters. *Estuar Coast Shelf S* **56**, 73-84, (2003).
114 Vieira, J. P. Ecological analogies between estuarine bottom trawl fish assemblages from Patos Lagoon, Rio Grande do Sul, Brazil and York River, Virginia, USA. *Rev Bras Zool* **23**, 234-247, (2006).
115 Greenwood, M. F. D., Matheson Jr., R. E., McMichael Jr., R. H. & MacDonald, T. C. Community structure of shoreline nekton in the estuarine portion of the Alafia River, Florida: Differences along a salinity gradient and inflow-related changes. *Estuar Coast Shelf S* **74**, 223-238, (2007).
116 Furey, N. B. & Sulikowski, J. A. The Fish Assemblage Structure of the Saco River Estuary. *Northeastern Naturalist* **18**, 37-44, (2011).
117 Wingate, R. L. & Secor, D. H. Effects of Winter Temperature and Flow on a Summer-Fall Nursery Fish Assemblage in the Chesapeake Bay, Maryland. *T Am Fish Soc* **137**, 1147-1156, (2008).
118 Able, K. W., Nemerson, D. M., Bush, R. & Light, P. Spatial Variation in Delaware Bay (U.S.A.) Marsh Creek Fish Assemblages. *Estuaries* **24**, 441-452, (2001).
119 Methven, D. A., Haedrich, R. L. & Rose, G. A. The Fish Assemblage of a Newfoundland Estuary: Diel, Monthly and Annual Variation. *Estuar Coast Shelf S* **52**, 669-687, (2001).
120 Bilkovic, D. M., Hershner, C. H. & Angstadt, K. Ecosystem approaches to aquatic health assessment: linking subtidal habitat quality, shoreline condition and estuarine fish communities. Final Report to NOAA/ NOAA Chesapeake Bay Office. 50 (Gloucester Point, Virginia, 2006).
121 Akin, S., Winemiller, K. O. & Gelwick, F. P. Seasonal and spatial variations in fish and macrocrustacean assemblage structure in Mad Island Marsh estuary, Texas. *Estuar Coast Shelf S* **57**, 269-282, (2003).
122 Baltz, D. M., Rakocinski, C. & Fleeger, J. W. Microhabitat use by marsh-edge fishes in a Louisiana estuary. *Environ Biol Fish* **36**, 109-126, (1993).
123 Fraser, T. H. Abundance, seasonality, community indices, trends and relationships with physicochemical factors of trawled fish in Upper Charlotte Harbor, Florida. *Bull Mar Sci* **60**, 739-763, (1997).
124 Baltz, D. M. & Jones, R. F. Temporal and Spatial Patterns of Microhabitat Use by Fishes and Decapod Crustaceans in a Louisiana Estuary. *T Am Fish Soc* **132**, 662-678, (2003).
125 Rozas, L. P., Minello, T. J., Zimmerman, R. J. & Caldwell, P. Nekton populations, long-term wetland loss, and the effect of recent habitat restoration in Galveston Bay, Texas, USA. *Mar Ecol Prog Ser* **344**, 119-130, (2007).
126 Moyle, P. B., Daniels, R. A., Herbold, B. & Baltz, D. M. Patterns in distribution and abundance of a noncoevolved assemblage of estuarine fishes in California. *Fish Bull* **84**, 105-117, (1986).
127 Stunz, G. W., Minello, T. J. & Rozas, L. P. Relative value of oyster reef as habitat for estuarine nekton in Galveston Bay, Texas. *Mar Ecol Prog Ser* **406**, 147-159, (2010).
128 Martino, E. J. & Able, K. W. Fish assemblages across the marine to low salinity transition zone of a temperate estuary. *Estuar Coast Shelf S* **56**, 969-987, (2003).
129 Hurst, T. P., McKown, K. A. & Conover, D. O. Interannual and Long-term Variation in the Nearshore Fish Community of the Mesohaline Hudson River Estuary. *Estuaries* **27**, 659-669, (2004).
130 Lazzari, M. A. Epibenthic Fishes and Decapod Crustaceans in Northern Estuaries: A Comparison of Vegetated and Unvegetated Habitats in Maine. *Estuaries* **25**, 1210-1218, (2002).
131 Granados-Dieseldorff, P. & Baltz, D. M. Habitat Use by Nekton along a Stream-Order Gradient in a Louisiana Estuary. *Estuaries Coasts* **31**, 572-583, (2008).
132 Rozas, L. P. & Minello, T. J. Nekton use of salt marsh, seagrass, and nonvegetated habitats in a South Texas (USA) estuary. *Bull Mar Sci* **63**, 481-501, (1998).
133 Weinstein, M. P. & Brooks, H. A. Comparative ecology of nekton residing in a tidal creek and adjacent seagrass meadow: community composition and structure. *Mar Ecol Prog Ser* **12**, 15-27, (1983).

134 Toft, J. D., Cordell, J. R., Simenstad, C. A. & Stamatiou, L. A. Fish Distribution, Abundance, and Behavior along City Shoreline Types in Puget Sound. *N Am J Fish Manage* **27**, 465-480, (2007).
135 Valle, C. F., O´Brien, J. W. & Wiese, K. B. Differential habitat use by California halibut, *Paralichthys californicus*, barred sand bass, *Paralabrax nebulifer*, and other juvenile fishes in Alamitos Bay, California. *Fish Bull* **97**, 646-660, (1999).
136 Szedlmayer, S. T. & Able, K. W. Patterns of Seasonal Availability and Habitat Use by Fishes and Decapod Crustaceans in a Southern New Jersey Estuary. *Estuaries* **19**, 697-709, (1996).
137 Nordby, C. S. & Zedler, J. B. Responses of Fish and Macrobenthic Assemblages to Hydrologic Disturbances in Tijuana Estuary and Los Peñasquitos Lagoon, California. *Estuaries* **14**, 80-93, (1991).
138 Ayvazian, S. G., Deegan, L. A. & Finn, J. T. Comparison of Habitat Use by Estuarine Fish Assemblages in the Acadian and Virginian Zoogeographic Provinces. *Estuaries* **15**, 368-383, (1992).
139 Allen, L. G. Seasonal abundance, composition, and productivity of the littoral fish assemblage in Upper Newport Bay, California. *Fish Bull* **80**, 769-790, (1982).
140 Horn, M. H. Diel and seasonal variation in abundance and diversity of shallow-water fish populations in Morro Bay, California. *Fish Bull* **78**, 759-770, (1980).
141 Allen, L. G. & Horn, M. H. Abundance, Diversity and Seasonality of Fishes in Colorado Lagoon, Alamitos Bay, California. *Estuar Coast Mar Sci* **3**, 371-380, (1975).
142 Bayer, R. D. Shallow-water Intertidal Ichthyofauna of the Yaquina Estuary, Oregon. *Northwest Science* **55**, 182-193, (1981).
143 Bottom, D. & Forsberg, B. The fishes of Tillamook Bay. 61 (Portland, Oregon, 1978).
144 Bottom, D. L., Miller, B. A. & Jones, K. K. A baseline survey of fish and invertebrates in the lower Umpqua River Estuary, Oregon. 86 (Portland, Oregon, 1985).
145 Cummings, T. E. & Berry, R. L. Some Observations on Fish Distribution in Tillamook Bay, Oregon, with Notes on Shellfish Temperature, and Physical Characteristics. 32pp. (Portland, Oregon, 1974).
146 Ellis, R. H. Tillamook Bay - Fish Use of the Estuary. 136pp. (Garibaldi, Oregon, 1999).
147 Ruiz, G. M., Hines, A. H. & Posey, M. H. Shallow water as a refuge habitat for fish and crustaceans in non-vegetated estuaries: an example from Chesapeake Bay. *Mar Ecol Prog Ser* **99**, 1-16, (1993).
148 Fresh, K. L. *Distribution and Abundance of Fishes Occuring in the Nearshore Surface Waters of Northern Puget Sound* MSc Thesis thesis, University of Washington. Washington State Department of Ecology Publication No. 01-06-030, Padilla Bay National Estuarine Research Reserve Reprint Series No. 36, (1979).
149 Allen, L. G., Findlay, A. M. & Phalen, C. M. Structure and Standing Stock of the Fish Assemblages of San Diego Bay, California from 1994 to 1999. *Bull South Calif Acad Sci* **101**, 49-85, (2002).
150 Williams, G. D., West, J. M. & Zedler, J. B. Shifts in Fish and Invertebrate Assemblages of Two Southern California Estuaries during the 1997-98 El niño. *Bull South Calif Acad Sci* **100**, 212-237, (2001).
151 Williams, G. D. & Zedler, J. B. Fish Assemblage Composition in Constructed and Natural Tidal Marshes of San Diego Bay: Relative Influence of Channel Morphology and Restoration History. *Estuaries* **22**, 702-716, (1999).
152 Peterson, M. S. & Ross, S. T. Dynamics of Littoral Fishes and Decapods along a Coastal River-Estuarine Gradient. *Estuar Coast Shelf S* **33**, 467-483, (1991).
153 Dahlberg, M. D. & Odum, E. P. Annual Cycles of Species Occurrence, Abundance, and Diversity in Georgia Estuarine Fish Populations. *American Midland Naturalist* **83**, 382-392, (1970).
154 Merriner, J. V., Kriete, W. H. & Grant, G. C. Seasonality, Abundance, and Diversity of Fishes in the Piankatank River, Virginia (1970-1971). *Chesapeake Science* **17**, 238-245, (1976).
155 Rountree, R. A. Fauna of Polyhaline Subtidal Marsh Creeks in Southern New Jersey: Composition, Abundance and Biomass. *Estuaries* **15**, 171-185, (1992).
156 Crabtree, R. E. & Dean, J. M. The Structure of Two South Carolina Estuarine Tide Pool Fish Assemblages. *Estuaries* **5**, 2-9, (1982).

157 Garwood, R. S., Mulligan, T. J. & Bjorkstedt, E. P. Ichthyological Assemblage and Variation in a Northern California *Zostera marina* Eelgrass Bed. *Northwestern Naturalist* **94**, 35-50, (2013).

158 Dahlberg, M. D. An Ecological Study of Georgia Coastal Fishes. *Fish Bull* **70**, 323-353, (1972).

159 Gorecki, R. & Davis, M. B. Seasonality and Spatial Variation in Nekton Assemblages of the Lower Apalachicola River. *Southeastern Naturalist* **12**, 171-196, (2013).

160 Livingston, R. J. Diurnal and Seasonal Fluctuations of Organisms in a North Florida Estuary. *Estuar Coast Mar Sci* **4**, 373-400, (1976).

161 Mabe, M. W. *Biogeographic characterization of fishes from intertidal sandflats in Pamlico River, North Carolina* MSc Thesis thesis, East Carolina University, (2012).

162 Ross, S. W. & Epperly, S. P. in *Fish Community Ecology in Estuaries and Coastal Lagoons: Towards an Ecosystem Integration* (ed A. Yáñez-Arancibia) Ch. 10, 207-241 (UNAM Press México, 1985).

163 Kirby-Smith, W. W., Lebo, M. E. & Herrmann, R. B. Nekton Variations in Tributaries along a Hydrologically Modified North Carolina Estuary. *Estuaries* **24**, 59-68, (2001).

164 Wenner, E. L., Shealy Jr., M. H. & Sandifer, P. A. A Profile of the Fish and Decapod Crustacean Community in a South Carolina Estuarine System Prior to Flow Alteration. 22pp. (1982).

165 Serafy, J. E., Lindeman, K. C., Hopkins, T. E. & Ault, J. S. Effects of freshwater canal discharge on fish assemblages in a subtropical bay: field and laboratory observations. *Mar Ecol Prog Ser* **160**, 161-172, (1997).

166 Hook, J. H. Seasonal variation in relative abundance and species diversity of fishes in South Bay. *Contrib Mar Sci* **32**, 127-141, (1991).

167 Oviatt, C. A. & Nixon, S. W. The Demersal Fish of Narragansett Bay: an Analysis of Community Structure, Distribution and Abundance. *Estuar Coast Mar Sci* **1**, 361-378, (1973).

168 Brooks, A. J. *Factors Influencing the Structure of an Estuarine Fish Community: The Role of Interspecific Competition* PhD Thesis thesis, University of California, (1999).

169 Lewis, M. A., Goodman, L. R., Chancy, C. A. & Jordan, S. J. Fish Assemblages in Three Northwest Florida Urbanized Bayous before and after Two Hurricanes. *J Coastal Res* **27**, 35-45, (2011).

170 Solomon, J. J., Brodie, R. B. & Ehlinger, G. S. Distribution and abundance of fish assemblages and select macroinvertebrates from the lower St. Marys river basin in Northeast Florida. *Florida Scientist* **69**, 1-18, (2006).

171 Cooley, N. R. An Inventory of the Estuarine Fauna in the Vicinity of Pensacola, Florida. 124 (Florida Department of natural Resources Marine Research Laboratory, St. Pertersburg, Florida, 1978).

172 Ogren, L. H. & Brusher, H. A. The distribution and abundance of fishes caught with a trawl in the St. Andrew Bay system , Florida. *Northeast Gulf Science* **1**, 83-105, (1977).

173 Melanson, R. L. & Campbell, C. E. Near-Shore Fish Populations within St. Pauls Inlet, an Estuarine System in Western Newfoundland. *Northeastern Naturalist* **19**, 487-500, (2012).

174 Currie, J. J., Wroblewski, J. S., Methven, D. A. & Hooper, R. G. The Nearshore Fish Fauna of Bonne Bay, a Fjord within Gros Morne National Park, Newfoundland. 66pp. (2009).

175 Shealy Jr., M. H., Miglarese, J. V. & Joseph, E. B. Bottom fishes of South Carolina estuaries - Relative abundance, seasonal distribution, and lenght-frequency relationships. 204pp. (1974).

176 Rulifson, R. A. Finfish Utilization of Man-Initiated and Adjacent Natural Creeks of South Creek Estuary, North Carolina Using Multiple Gear Types. *Estuaries* **14**, 447-464, (1991).

177 Purvis, C. Nursery Area Survey of Northern Pamlico Sound and Tributaries. Completion Report for Project Number 2-230-R. 66pp. (N. C. Division of Marine Fisheries, Morehead City, 1976).

178 Fiske, J. D., Curley, J. R. & Lawton, R. P. A study of the marine resources of the Westport River. 59pp. (1968).

179 Meng, L. & Powell, J. C. Linking Juvenile Fish and Their Habitats: An Example from Narragansett Bay, Rhode Island. *Estuaries* **22**, 905-916, (1999).

180 Moore, R. H. Variations in the Diversity of Summer Estuarine Fish Populations in Aransas Bay, Texas, 1966-1973. *Estuar Coast Mar Sci* **6**, 495-501, (1978).
181 Targett, T. E. & McCleave, J. Summer Abundance of Fishes in a Maine Tidal Cove With Special Reference to Temperature. *T Am Fish Soc* **103**, 325-330, (1974).
182 Lazzari, M. A. & Tupper, B. Importance of shallow water habitats for demersal fishes and decapod crustaceans in Penobscot Bay, Maine. *Environ Biol Fish* **63**, 57-66, (2002).
183 Robards, M. D., Piatt, J. F., Kettle, A. B. & Abookire, A. A. Temporal and geographic variation in fish communities of lower Cook Inlet, Alaska. *Fish Bull* **97**, 962-977, (1999).
184 Hillman, R. E., Davis, N. W. & Wennemer, J. Abundance, Diversity, and Stability in Shore-zone Fish Communities in an Area of Long Island Sound Affected by the Thermal Discharge of a Nuclear Power Station. *Estuar Coast Mar Sci* **5**, 355-381, (1977).
185 Rosales-Casián, J. A. Inshore soft-bottom fishes of two coastal lagoons on the Northern Pacific coast of Baja California. *CalCOFI Rep.* **38**, 180-192, (1997).
186 Subrahmanyam, C. B. & Coultas, C. L. Studies on the animal communities in two North Florida salt marshes, Part III. Seasonal fluctuations of fish and macroinvertebrates. *Bull Mar Sci* **30**, 790-818, (1980).
187 Jennings, C. A. & Weyers, R. S. Temporal and Spatial Distribution of Estuarine-Dependent Species in the Savannah River Estuary, July 2000 - December 2002. Project Final Report. 183pp. (Savannah, Georgia, 2003).
188 Kimmerer, W. J., Gross, E. S. & MacWilliams, M. L. Is the Response of Estuarine Nekton to Freshwater Flow in the San Francisco Estuary Explained by Variation in Habitat Volume? *Estuaries Coasts* **32**, 375-389, (2009).
189 Garcia, A. M. & Vieira, J. P. O aumento da diversidade de peixes no estuário da Lagoa dos Patos durante o episódio El Niño 1997-1998. *Atlântica* **23**, 85-96, (2001).
190 Ramos, L. A. & Vieira, J. P. Species composition and abundance of shallow water fishes of five estuaries of Rio Grande do Sul State, Brazil. *Boletim do Instituto de Pesca* **27**, 109-121, (2001).
191 Jaureguizar, A. J., Menni, R., Guerrero, R. & Lasta, C. Environmental factors structuring fish communities of the Río de la Plata estuary. *Fish Res* **66**, 195-211, (2004).
192 Barletta, M., Barletta-Bergan, A., Saint-Paul, U. & Hubold, G. The role of salinity in structuring the fish assemblages in a tropical estuary. *J Fish Biol* **66**, 45-72, (2005).
193 Loebmann, D. & Vieira, J. P. Composição e abundância dos peixes do Parque Nacional da Lagoa do Peixe, Rio Grande do Sul, Brasil e comentários sobre a fauna acompanhante de crustáceos decápodos. *Atlântica* **27**, 131-137, (2005).
194 Vendel, A. L. & Chaves, P. d. T. Use of an estuarine environment (Barra do Saí lagoon, Brazil) as nursery by fish. *Rev Bras Zool* **23**, 1117-1122, (2006).
195 López-López, E., Elías Sedeño-Díaz, J., Romero, F. L. & Trujillo-Jiménez, P. Spatial and seasonal distribution patterns of fish assemblages in the Río Champotón, southeastern Mexico. *Rev Fish Biol Fisher* **19**, 127-142, (2009).
196 de Paiva, A. C. G., Lima, M. F. V., de Souza, J. R. B. & de Araújo, M. E. Spatial distribution of the estuarine ichthyofauna of the Rio Formoso (Pernambuco, Brazil), with emphasis on reef fish. *Zoologia* **26**, 266-278, (2009).
197 Vilar, C. C. *et al.* Local and regional ecological drivers of fish assemblages in Brazilian estuaries. *Mar Ecol Prog Ser* **485**, 181-197, (2013).
198 Mendoza, E., Castillo-Rivera, M., Zárate-Hernández, R. & Ortiz-Burgos, S. Seasonal variations in the diversity, abundance, and composition of species in an estuarine fish community in the Tropical Eastern Pacific, Mexico. *Ichthyol Res* **56**, 330-339, (2009).
199 Contente, R. F., Stefanoni, M. F. & Spach, H. L. Fish assemblage structure in an estuary of the Atlantic Forest biodiversity hotspot (southern Brazil). *Ichthyol Res* **58**, 38-50, (2011).
200 Vendel, A. L., Bouchereau, J.-L. & Chaves, P. d. T. Environmental and Subtidal Fish Assemblage Relationships in Two Different Brazilian Coastal Estuaries. *Braz arch biol technol* **53**, 1393-1406, (2010).
201 Chaves, P. & Bouchereau, J.-L. Biodiversité et dynamique des peuplements ichtyiques de la mangrove de Guaratuba, Brésil. *Oceanol Acta* **22**, 353-364, (1999).
202 Rueda, M. & Defeo, O. Spatial structure of fish assemblages in a tropical estuarine lagoon: combining multivariate and geostatistical techniques. *J Exp Mar Biol Ecol* **296**, 93-112, (2003).

203 Loebmann, D. & Vieira, J. P. O impacto da pesca do camarão-rosa *Farfantepenaeus paulensis* (Perez-Farfante) (Decapoda, Penaeidae) nas assembléias de peixes e siris do Parque Nacional da Lagoa do Peixe, Rio Grande do Sul, Brasil. *Rev Bras Zool* **23**, 1016-1028, (2006).
204 Araújo, F. G., da Cruz-Filho, A. G., de Azevêdo, M. C. C. & Santos, A. C. d. A. Estrutura da comunidade de peixes demersais da Baía de Sepetiba, RJ. *Rev Bras Biol* **58**, 417-430, (1998).
205 Castillo-Rivera, M., Zavala-Hurtado, J. A. & Zárate, R. Exploration of spatial and temporal patterns of fish diversity and composition in a tropical estuarine system of Mexico. *Rev Fish Biol Fisher* **12**, 167-177, (2002).
206 Castillo-Rivera, M., Ortiz-Burgos, S. & Zárate-Hernández, R. Estuarine fish community in a submerged vegetation habitat: seasonal and diel variations. *Hidrobiológica* **21**, 311-321, (2011).
207 González Castro, M. *et al.* Fish composition in a south-western Atlantic temperate coastal lagoon: spatial–temporal variation and relationships with environmental variables. *J Mar Biol Assoc UK* **89**, 593-604, (2009).
208 Loebmann, D. *et al.* Composition and Relative Abundance of Fish Species in Two Coastal Lagoons in Austral South America: Peixe Lagoon (31ºS; 51ºW), Brazil, and Mar Chiquita Lagoon (37ºS; 57ºW), Argentina. *Neotrop Biol Conserv* **3**, 28-33, (2008).
209 Ramos-Miranda, J. *et al.* Spatial and temporal changes in the nekton of the Terminos Lagoon, Campeche, Mexico. *J Fish Biol* **66**, 513-530, (2005).
210 Neves, L. M., Teixeira, T. P. & Araújo, F. G. Structure and dynamics of distinct fish assemblages in three reaches (upper, middle and lower) of an open tropical estuary in Brazil. *Mar Ecol* **32**, 115-131, (2011).
211 Ramírez, C. S. & Rueda, M. Variación de la diversidad y abundancia de especies ícticas dominantes en el Delta del Río Magdalena, Colombia. *Rev. Biol. Trop.* **47**, 1067-1079, (1999).
212 Raz-Guzman, A. & Huidobro, L. Fish communities in two environmentally different estuarine systems of Mexico. *J Fish Biol* **61**, 182-195, (2002).
213 Vilar, C. C., Spach, H. L. & Souza-Conceição, J. M. Fish assemblage in shallow areas of Baía da Babitonga, southern Brazil: structure, spatial and temporal patterns. *Pan-Am J Aquat Sci* **6**, 303-319, (2011).
214 Vilar, C. C., Spach, H. L. & Joyeux, J. C. Spatial and temporal changes in the fish assemblage of a subtropical estuary in Brazil: environmental effects. *J Mar Biol Assoc UK* **91**, 635-648, (2011).
215 Vilar, C. C., Spach, H. L. & Santos, L. d. O. Fish fauna of Baía da Babitonga (southern Brazil), with remarks on species abundance, ontogenic stage and conservation status. *Zootaxa* **2734**, 40-52, (2011).
216 Spach, H. L. *et al.* Assembleias de peixes em diferentes ambientes da desembocadura do Rio Saí Guaçu, Sul do Brasil. *Pan-Am J Aquat Sci* **5**, 126-138, (2010).
217 Schwarz Jr., R. *et al.* Composition and structure of the demersal ichthyofauna in the Pinheiros Bay, Paraná, Brazil. *BJAST* **10**, 27-39, (2006).
218 Santos, C., Schwarz Jr., R., de Oliveira Neto, J. F. & Spach, H. L. The ichthyofauna in two tidal flats of the euhaline section of Paranaguá Bay, PR. *Boletim do Instituto de Pesca, São Paulo* **28**, 49-60, (2002).
219 Reis-Filho, J. A., Nunes, L. D. C., de Menezes, B. L. & de Souza, G. B. G. Variação espaço-temporal e efeito do ciclo lunar na ictiofauna estuarina: evidências para o estuário do Rio Joanes - Bahia. *Biotemas* **23**, 111-122, (2010).
220 de Oliveira-Silva, J. T., Peso-Aguiar, M. C. & Lopes, P. R. D. Ictiofauna das praias de Cabuçu e Berlinque: Uma contribuição ao conhecimento das comunidades de peixes na Baía de Todos os Santos - Bahia - Brasil. *Biotemas* **21**, 105-115, (2008).
221 Lima, M. A. T. *Composição da ictiofauna demersal do estuário do Rio de Contas, Bahia, Brasil* MSc Thesis thesis, Universidade Estadual de Santa Cruz, (2010).
222 Falcão, M. G. *et al.* A ictiofauna em planícies de maré das Baías das Laranjeiras e de Paranaguá, Paraná, Brasil. *Zoociências* **8**, 125-138, (2006).
223 de Oliveira, A. M. E. Composição e distribuição da ictiofauna, nas águas estuarinas do Rio Jaguaribe (Ceará - Brasil). *Arquivos de Ciencias do Mar* **16**, 9-18, (1976).
224 Arceo-Carranza, D., Vega-Cendejas, M. E., Montero-Muñoz, J. L. & de Santillana, M. J. H. Influence of habitat type on diel fish associations in a tropical coastal lagoon. *Rev Mex Biodivers* **81**, 823-837, (2010).

225 Feutry, P., Hartmann, H. J., Casabonnet, H. & Umaña, G. Preliminary analysis of the fish species of the Pacific Central American Mangrove of Zancudo, Golfo Dulce, Costa Rica. *Wetl Ecol Manag* **18**, 637-650, (2010).
226 Phillips, P. C. Diversity and fish community structure in a Central American mangrove embayment. *Rev. Biol. Trop.* **29**, 227-236, (1981).
227 Hercos, A. P. *Diversidade e variabilidade espaço-temporal da ictiofauna da região estuarina do rio Curuçá Município de Curuçá, Pará Brasil* MSc Thesis thesis, Universidade Federal do Pará, (2006).
228 Amezcua-Linares, F. Generalidades ictiológicas del sistema lagunar costero de Huizache-Caimanero, Sinaloa, México. *Anales del Centro de Ciencias del Mar y Limnología* **4**, 1-26, (1976).
229 Warburton, K. Community Structure, Abundance and Diversity of Fish in a Mexican Coastal Lagoon System. *Estuar Coast Mar Sci* **7**, 497-519, (1978).
230 Yáñez-Arancibia, A. in *39th Annual Meeting American Society of Limnology and Oceanography* 302 pp. (Publicaciones Especiales Centro de Ciencias del Mar y Limnología, Savannah, Georgia, 1978).
231 Rodrigues, C., Lavrado, H. P., Falcão, A. P. d. C. & da Silva, S. H. G. Distribuição da ictiofauna capturada em arrastos de fundo na Baía de Guanabara - Rio de Janeiro, Brasil. *Arquivos do Museu Nacional, Rio de Janeiro* **65**, 199-210, (2007).
232 Cartagena, B. F. C., Hostim-Silva, M. & Spach, H. L. Spatial distribution of demersal fish in the district of Saco dos Limões (Baía Sul, Florianópolis, state of Santa Catarina, Brazil). *Bioikos, Campinas* **25**, 117-128, (2011).
233 Pessanha, A. L. M. & Araújo, F. G. Spatial, temporal and diel variations of fish assemblages at two sandy beaches in the Sepetiba Bay, Rio de Janeiro, Brazil. *Estuar Coast Shelf S* **57**, 817-828, (2003).
234 Hackradt, C. W. *et al.* A estrutura da comunidade de peixes em praias de baixa energia do complexo estuarino da Baía de Paranaguá, Brasil. *Zoociências* **11**, 233-244, (2009).
235 Álvarez-León, R. Ictiofauna del complejo fluvio-lagunar-estuarino de la Ciénaga Grande de Santa Marta (Colombia), antès de las obras civiles de recuperación de los caños. *DAHLIA - Revista de la Asociación Colombiana de Ictiólogos* **6**, 79-90, (2003).
236 Bartels, C. E., Price, K. S., López, M. I. & Bussing, W. A. Occurrence, distribution, abundance and diversity of fishes in the Gulf of Nicoya, Costa Rica. *Rev. Biol. Trop.* **31**, 75-101, (1983).
237 Gonzalez, R. C. *Composicion y abundancia de la ictiofauna del estero de "El Verde", Sinaloa* Bachelor Thesis thesis, Instituto Politecnico Nacional, (1980).
238 Diaz-Ruiz, S., Pérez-Hernández, M. A. & Aguirre-León, A. Characterization of fish assemblages in a tropical coastal lagoon in the northwest Gulf of Mexico. *Cienc Mar* **29**, 631-644, (2003).
239 Díaz-Ruiz, S., Cano-Quiroga, E., Aguirre-León, A. & Ortega-Bernal, R. Diversidad, abundancia y conjuntos ictiofaunísticos del sistema lagunar-estuarino Chantuto-Panzacola, Chiapas, México. *Rev. Biol. Trop.* **52**, 187-199, (2004).
240 Franco-López, J. & Chavez-López, R. Sintesis sobre el conocimiento de la ictiofauna de la Laguna de Tamiahua, Veracruz, Mexico. *Hidrobiológica* **3/4**, 53-63, (1992).
241 León, P. E. Ecología de la ictiofauna del Golfo de Nicoya, Costa Rica, un estuario tropical. *Rev. Biol. Trop.* **21**, 5-30, (1973).
242 León, R. A. Necton y bentos de tres esteros adyacentes a Mazatlán (Sin.) México. *Rev. Biol. Trop.* **28**, 237-262, (1980).
243 Tapia-García, M., Núñez, C. S., de Guevara, G. C. L., Montes, M. C. M. & Abad, M. C. G. Composición y distribución de la ictiofauna en la Laguna del Mar Muerto, Pacífico mexicano. *Rev. Biol. Trop.* **46**, 277-284, (1998).
244 Núñez, C. S. *Caracterizacion de las comunidades de peces de la Laguna del Mar Muerto Oaxaca-Chiapas*, Universidad Autonoma Metropolitana, (1993).
245 Vera, L. A. & Muñoz, J. d. l. R. Estructura de la comunidad íctica de la ciénaga de Mallorquín, Caribe Colombiano. *Boletín de Investigaciones Marinas y Costeras* **32**, 231-242, (2003).
246 Alves, M. I. M. & Filho, A. A. S. Peixes do estuário do Rio Jaguaribe (Ceará - Brasil): aspectos fisioecológicos. *Ciência Agronómica* **27**, 5-16, (1996).
247 Claus, S. *et al.* MarineRegions.org, http://www.marineregions.org, (2016) (Date of access 01/01/2016)
